# Genome-wide identification and characterization of the *ALOG* gene family in *Petunia*

**DOI:** 10.1186/s12870-019-2127-x

**Published:** 2019-12-30

**Authors:** Feng Chen, Qin Zhou, Lan Wu, Fei Li, Baojun Liu, Shuting Zhang, Jiaqi Zhang, Manzhu Bao, Guofeng Liu

**Affiliations:** 1 Key Laboratory of Horticultural Plant Biology, Ministry of Education, College of Horticulture and Forestry Sciences, Huazhong Agricultural University, Shizishan Street No. 1, Wuhan, 430070 China; 2 Guangzhou Institute of Forestry and Landscape Architecture, Guangzhou, 510405 China

**Keywords:** Petunia, *ALOG* genes, DUF640 domain, Transcription factor, Expression pattern, Plant development

## Abstract

**Background:**

The ALOG (*Arabidopsis* LSH1 and *Oryza* G1) family of proteins, namely DUF640 (domain of unknown function 640) domain proteins, were found in land plants. Functional characterization of a few ALOG members in model plants such as *Arabidopsis* and rice suggested they play important regulatory roles in plant development. The information about its evolution, however, is largely limited, and there was no any report on the *ALOG* genes in *Petunia*, an important ornamental species.

**Results:**

The *ALOG* genes were identified in four species of *Petunia* including *P. axillaris*, *P. inflata*, *P. integrifolia,* and *P. exserta* based on the genome and/or transcriptome databases, which were further confirmed by cloning from *P. hybrida* ‘W115’ (Mitchel diploid), a popular laboratorial petunia line susceptible to genetic transformation. Phylogenetic analysis indicated that *Petunia ALOG* genes (named as *LSHs* according to their closest *Arabidopsis* homologs) were grouped into four clades, which can be further divided into eight groups, and similar exon**-**intron structure and motifs are reflected in the same group. The *PhLSH* genes of hybrid petunia ‘W115’ were mainly derived from *P. axillaris*. The qPCR analysis revealed distinct spatial expression patterns among them suggesting potentially functional diversification. Moreover, over-expressing *PhLSH7a* and *PhLSH7b* in *Arabidopsis* uncovered their functions in the development of both vegetative and reproductive organs.

**Conclusions:**

Petunia genome includes 11 *ALOG* genes that can be divided into eight distinct groups, and they also show different expression patterns. Among these genes, *PhLSH7b* and *PhLSH7a* play significant roles in plant growth and development, especially in fruit development. Our results provide new insight into the evolution of *ALOG* gene family and have laid a good foundation for the study of petunia *LSH* gene in the future.

## Background

Transcription factors (TFs) are involved in activation and/or inhibition of transcription in respond to a variety of endogenous and environmental signals, and play a crucial role in the regulation of many developmental processes and defensive responses in plants. According to the regulatory function, mechanism of action, and the sequence homology of their DNA binding domains (DBD) and other conserved motifs, TFs can be divided into different categories [1]. Usually, there are two mechanistic classes of transcription factors: basal and specific TFs [2]. Different from the highly conserved basal TFs, which are ubiquitous in all organism and necessary for transcription to occur, specific TFs have great diversity in structure and phyletic distribution [3]. For example, ALOG-domain proteins, a family of plant specific TFs, controls plant growth and development in many aspects [4–9].

The ALOG family was named after *Arabidopsis* LSH1 and *Oryza* G1, the first two family members identified from eudicots and monocots, respectively. A highly conserved domain corresponding to DUF640 in the protein-family (Pfam) database (Pfam accession number IPR006936) was identified in ALOG family [4, 5]. DUF families encode functionally uncharacterized proteins, but the ALOG/DUF640 proteins had been suggested to function as specific TFs in plant based on the characteristics of sequence-specific DNA binding, transcriptional regulatory activity, nuclear location, and homodimer formation [5, 7, 10, 11]. With 4 all-α helices and additional insertion of a zinc ribbon, ALOG domain was predicted to be a N-terminal DNA-binding domain originated from the XerC/D-like recombinases of a new category of DIRS-1-like retroposons [10]. Furthermore, ALOG-like domains also exist in certain plant defense proteins, where they may function as DNA sensor [10, 12].

Up to now, our knowledge on the evolution and function of ALOG proteins is very limited. Only a few genes have been studied in the ALOG family, which indicate they play a key role in developmental regulation [4–8, 13, 14]. *AtLSH1* was the first *ALOG* gene identified in the plant kingdom, which was cloned from a dominant mutant (*lsh1-D*) of *Arabidopsis* that displays hypersensitive to uninterrupted far-red, blue and red light and produces shorter hypocotyl than wild-type plants. *AtLSH1* mediates light regulation during seedling development, which functionally depends on phytochromes [4]. Recent studies have also revealed the function of ALOG domain genes in the establishment of organ boundaries [7, 15]. For instance, *AtLSH3* (*ORGAN BOUNDARY1, OBO1*) and *AtLSH4* (*OBO4*) genes are expressed in the boundary cells of various lateral organs, including cotyledons, leaves and floral organs, which is directly up-regulated by CUC1 (CUP-SHAPED COTYLEDON1), a NAC protein that plays central roles in embryonic SAM (shoot apical meristems) formation and specification of the shoot organ boundaries [7, 16, 17]. Over-expressing *OBO1* disrupts numbers and size of petals and causes petal-stamen fusion, while ablation of the *OBO1*-expressing cells leads to loss of the shoot apical meristems and lateral organs; ectopic expression of *OBO4* in the shoot apex results in inhibition of leaf development and formation of extra shoots or shoot organs within flowers [7, 15, 16]. These results indicate that *AtLSH3* and *AtLSH4* may inhibit organ differentiation in the boundary regions [16–18].

Studies in other species revealed that ALOG family proteins mediate the regulation of inflorescence architecture and flower organ development. *Long sterile lemma* **(***G1*) specifies the identity of sterile lemma in rice by repressing the homeotic transformation of the sterile lemma to the regular lemma [5]. Another homolog of *OsG1* in rice, *TH1* (*TRIANGULAR HULL1*), also known as *BSG1* (*BEAK-SHAPED GRAIN1*)/*BLS1* (*BEAK LIKE SPIKELET1*)/*BH1* (*BEAK-SHAPED HULL1*)/*AFD1* (*ABNORMAL FLOWER AND DWARF1*), regulate cell extension of the lemma and palea to determine grain shape and size [11, 19–21], which may function as a transcription repressor and regulate downstream hormone signal transduction and starch/sucrose metabolism related genes [11, 20, 21]. Contrast to *G1* and *TH1*, *TAWAWA1 (TAW1)*, the third rice *ALOG* gene, is a specific regulator of meristems activity and regulates inflorescence architecture by maintaining an indeterminate fate of inflorescence meristems and inhibiting the phase change to spikelet meristems identity, probably through up-regulating *SVP-like* genes such as *OsMADS22*, *OsMADS47*, and *OsMADS55* [6, 22]. Similarly, an ALOG protein in tomato (*Solanum lycopersicum*), TERMINATING FLOWER (TMF), affects inflorescence organization by suppressing the inflorescence meristems to adopt floral fate [8]. In the *tmf* mutant, the primary SAMs terminate as single flowers, instead of forming sympodial inflorescence meristems or vegetative shoot meristems, which is partly due to precocious expression of *FALSIFLORA (FA)* and *ANANTHA (AN)*, the orthologous genes of *Arabidopsis LFY* and *UFO*, respectively [8].

Petunia is an important ornamental species and an ideal model for comparative study of gene functions attributed to the characteristics of highly efficient genetic transformation, easy of cultivation and propagation [23–25]. Considering the limitation of evolution and function of ALOG proteins, petunia can be an ideal material to conduct in-depth study. Recently, genome sequencing was accomplished in *P. inflata* and *P. axillaris* [26], which offer an effective basis to investigate the gene family on a genome-wide scale. In this study, the first characterization of the entire *ALOG* gene family was performed in *Petunia*, including the analysis of gene structure, motif composition, phylogenetic classification and expression pattern. As a result, we identified 11 ALOG domain genes in *Petunia* and confirmed these genes by cloning from *P. hybrida* line W115. Multiple alignments between *PhLSHs* and homologous genes from four wild *Petunia* species suggested that *P. axillaris* was the major paternal donor of *PhLSHs*. In addition, the function of *PhLSH7a* and *PhLSH7b* was characterized by over-expressing in *Arabidopsis* plants. The results have laid a solid foundation for further elucidating the functions of ALOG-domain genes in petunia.

## Results

### Identification of *ALOG* genes in *Petunia*

*Petunia ALOG* genes were identified by the HMM profile for the DUF640 domain (PF04852) search and tBLASTx (translated nucleotide to translated nucleotide) search with *Arabidopsis* and rice ALOG proteins against the genomic and transcriptomic databases of *P. inflata* and *P. axillaris* [27, 28], respectively. As a result, 11 *ALOG* genes were identified in *P. axillaris*, named *PaLSHs* (Additional file 1), and 13 *ALOG* genes were identified in *P. inflata*, named *PiLSHs* (Additional file 2). These genes were numbered based on their homologs in *Arabidopsis*. In *P. inflata*, the three *PiLSH5* genes have identical coding sequences, and so only one was used in the subsequent analysis.

To identify the complete open reading frame (ORF) and the exon-intron distribution of the *Petunia LSHs*, we designed the gene-specific primers (Additional file 3) to amplify the full-length coding sequences (CDS) and their genomic DNA (gDNA) sequences of *PhLSH*s from W115*.* Finally, all putative petunia *LSH* genes were isolated and confirmed by sequencing (Table 1).

**Table 1:** *LSH* genes in *P. hybrida* ‘W115’ (Mitchel diploid)

Gene name	NCBI Accession number	CDS length (bp)	Gene length (bp)	Protein length (aa)
*PhLSH1*	MK179267	585	1112	194
*PhLSH2*	MK179268	483	483	160
*PhLSH3a*	MK179269	594	976	197
*PhLSH3b*	MK179270	576	576	191
*PhLSH4*	MK179271	567	567	188
*PhLSH5*	MK179272	696	794	231
*PhLSH7a*	MK179273	582	582	193
*PhLSH7b*	MK179274	555	555	184
*PhLSH10a*	MK179275	540	540	179
*PhLSH10b*	MK179276	534	534	177
*PhLSH10c*	MK179277	516	516	171

### Sequence characteristics of *Petunia ALOG* genes

**Fig. 1:**
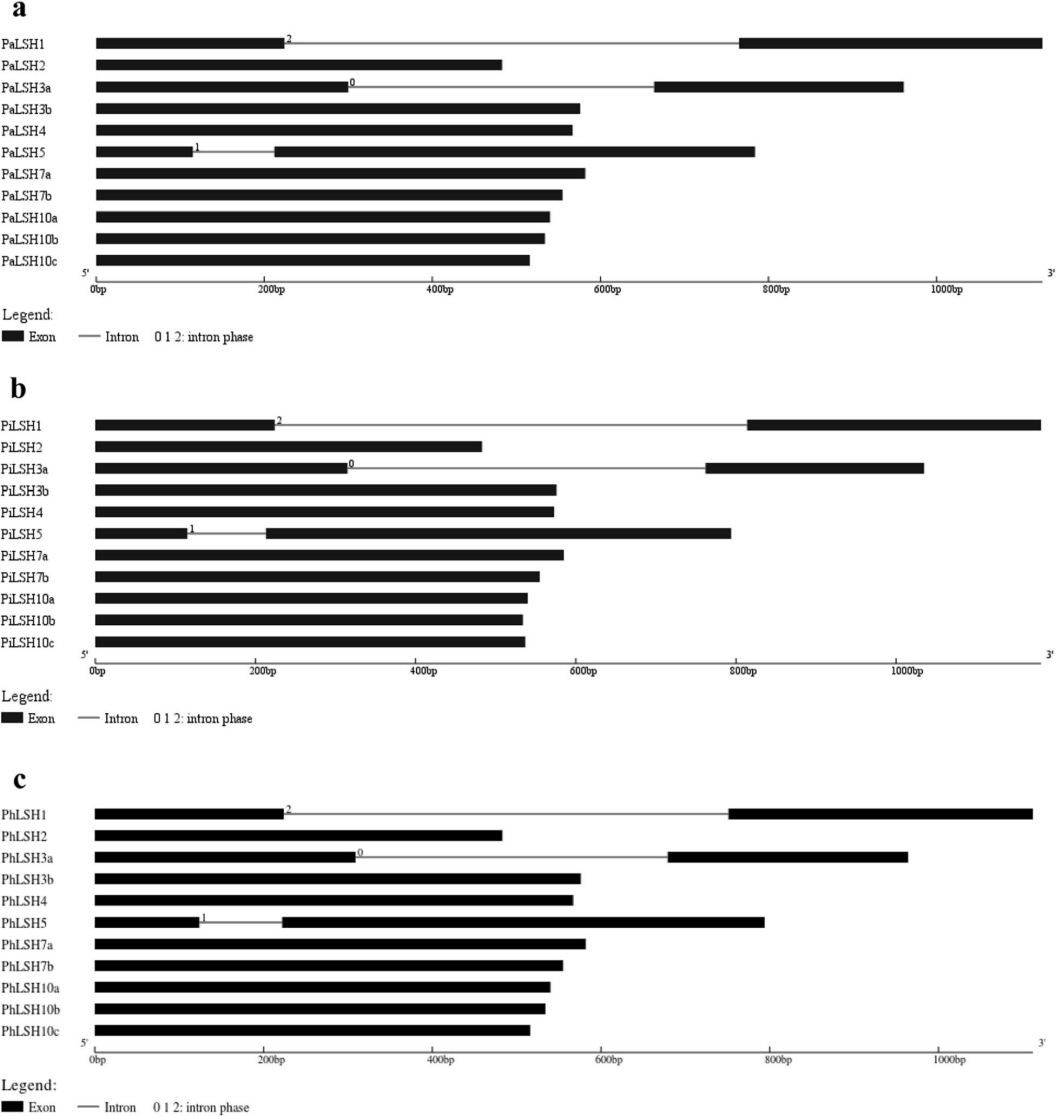
Gene structures of *PaLSHs* (**a**), *PiLSHs* (**b**) and *PhLSHs* (**c**). The black block indicates the exons, and the gray thin line indicates the introns. The intron phases were represented by 0, 1 and 2

Sequence analysis showed that the gene length of *Petunia LSHs* vary from 483 bp (*PaLSH2*) to 1126 bp (*PaLSH1*) in *P. axillaris* (Additional file 1), 483 bp (*PiLSH2*) to 1180 bp (*PiLSH1*) in *P. inflata* (Additional file 2), and 483 bp (*PhLSH2*) to 1112 bp (*PhLSH1*) in *P. hybrida* (Table 1). The CDS length of *PhLSHs* vary from 483 bp (*PhLSH2*) to 696 bp (*PhLSH5*), with corresponding length of PhLSH proteins from 160 to 231 aa (Table 1). Exon-intron analysis indicated that *PhLSH1*, *PhLSH3a*, and *PhLSH5* had two exons and one intron, while the other 8 genes only have a single exon without an intron (Fig. 1c), consistent with the results predicted by the genomic data of *PiLSHs* and *PaLSHs* (Fig. 1a, b).

### Phylogenetic analysis of PhLSH proteins

**Fig. 2:**
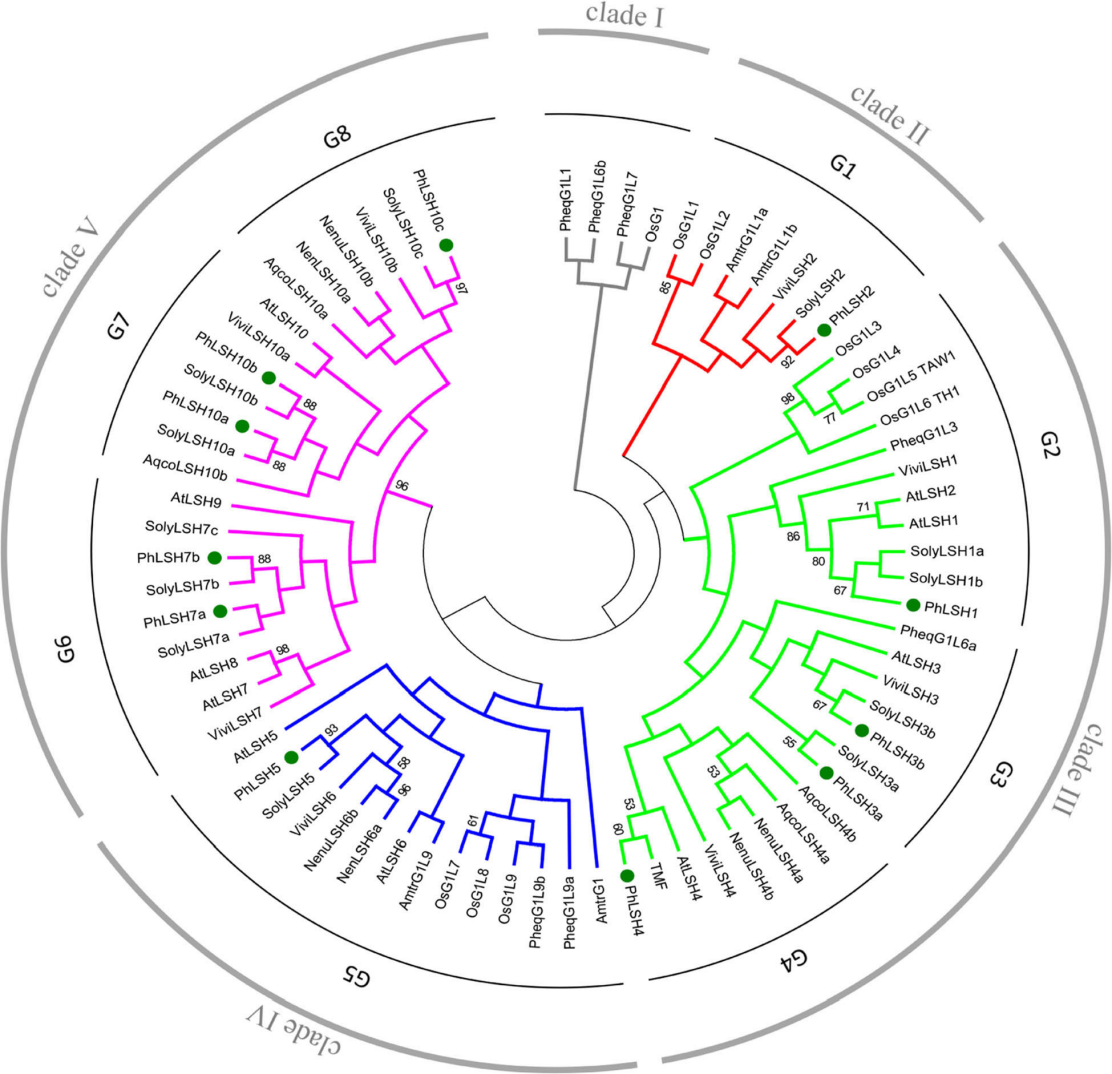
Phylogenetic tree of the ALOG proteins in different species. Phylogenetic analysis based on the conserved ALOG domains (130 aa) of a total of 73 protein sequences. Species abbreviations are as follows. Ph: *Petunia hybrida*; At: *Arabidopsis thaliana*; Os: *Oryza sativa*; Vivi: *Vitis vinifera*; Amtr: *Amborella trichopoda*; Phap: *Phalaenopsis aphrodite*; Nenu: *Nelumbo nucifera*; Aqco: *Aquilegia coerulea* and Soly: *Solanum lycopersicum*. The tree was generated by the Maximum likelihood (ML) method in MEGA 6.0 with 1000 bootstrap replicates. Letters outside of the tree indicate the defined groups

The phylogenetic relationship between *PhLSHs* and homologous genes in other species will contribute to reveal the evolution of ALOG family and gene function in petunia. Therefore, we constructed a Maximum Likelihood (ML) phylogenetic tree with the ALOG proteins identified from representative species within different clades of angiosperms (Additional file 4). As a result, the ALOG proteins were divided into five clades: clade I (OsG1-like) includes proteins only from monocots, clade II (OsG1L1/2-like) includes proteins from *Amborella trichopoda,* grape, tomato and petunia, clade III and IV (AtLSH1/2/3/4-like and AtLSH5/6-like, respectively) include protein from almost all species, while clade V (AtLSH7/8/9/10-like) only includes proteins from eudicots (Fig. 2). The petunia *LSH* genes were distributed in clade II-V, which can be further divided into 8 groups: Group 1 (G1) including *PhLSH2*; Group 2 (G2) including *PhLSH1*; Group 3 (G3) containing *PhLSH3a* and *PhLSH3b*; Group 4 (G4) containing *PhLSH4*; Group 5 (G5) containing *PhLSH5*; Group 6 (G6) containing *PhLSH7a* and *PhLSH7b*; Group 7 (G7) containing *PhLSH10a* and *PhLSH10b*, and Group 8 (G8) containing *PhLSH10c* (Fig. 2). Both the redundancy of function and divergence of the *LSH* gene family could be reflected in this classification. Lineage-specific duplication can be revealed from this phylogenetic tree. In plants, duplication of some *LSH* genes appears to occur very late. For example, duplication of genes in Group V and VI had occurred after the separation of the monocot and dicot.

### Origin of *PhLSH* genes

**Fig. 3:**
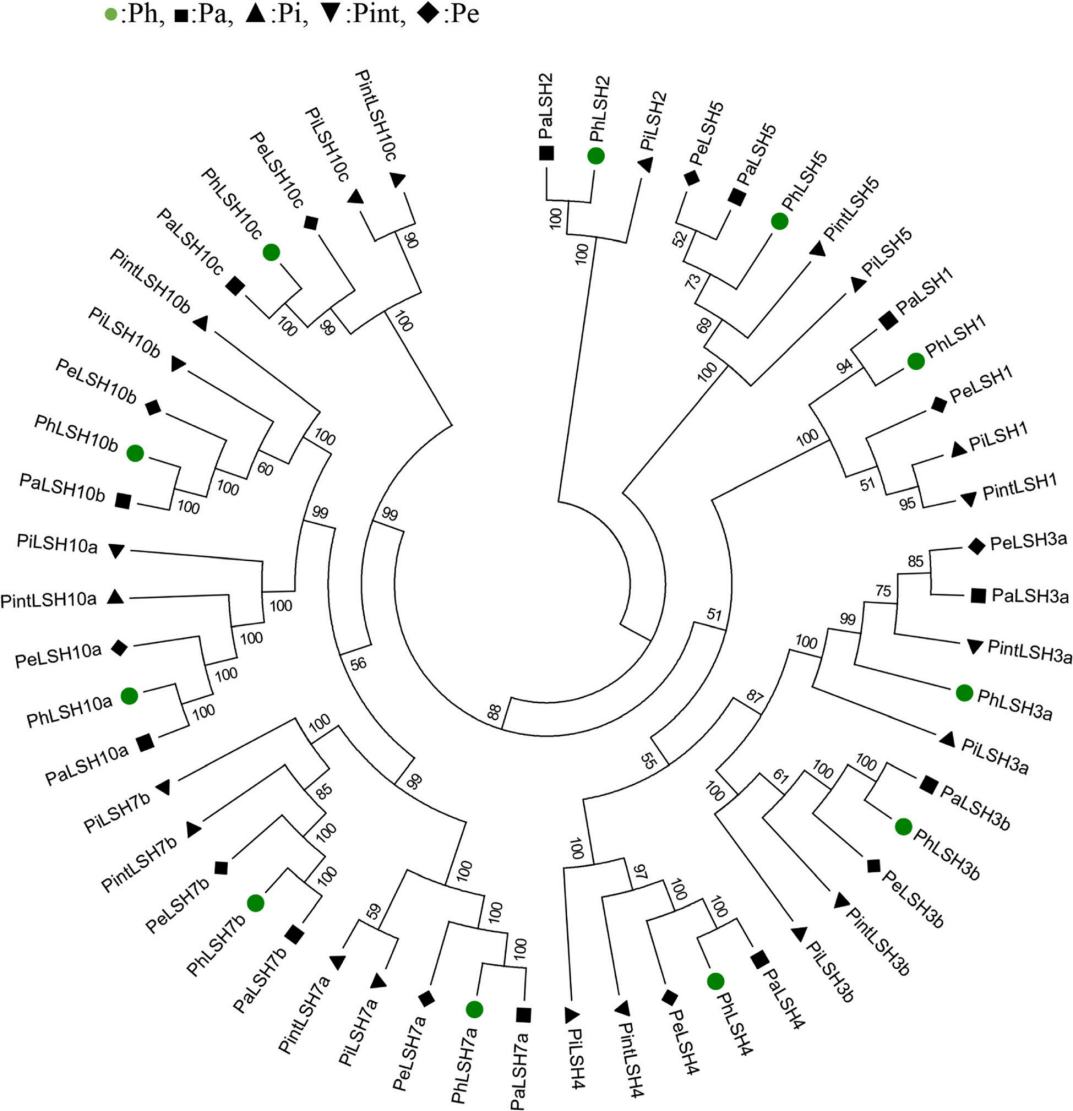
Phylogenetic tree for the CDS of petunia *LSHs*. *Ph*, *P. hybrida*; *Pa*, *P. axillaris*; *Pi*, *P. inflata*; *Pint*, *P. integrifolia*; and *Pe*, *P. exserta*. The tree was constructed by MEGA 6.0 using Maximum Likelihood method based on the GTR + G + I model with 1000 bootstrap replicates. Branches corresponding to partitions reproduced in less than 50% bootstrap replicates are collapsed

Multiple sequence alignment based on the CDS of *LSHs* in W115 and four *Petunia* wild species (*P. integrifolia*, *P. exserta*, *P. axillaris* and *P. inflata*) was used to determine the origin of *PhLSH* genes, i.e. which species was the donor of *PhLSH* genes. Only 10 of the 11 *LSH* genes could be identified from the Transcriptome Shotgun Assembly (TSA) database of *P. integrifolia* and *P. exserta* (Additional file 5) [﻿29﻿, 30]. The orthologs of *PhLSH2* are not recognized in *P. integrifolia* and *P. exserta* and so not included in the subsequent analysis. A phylogenetic relationship constructed with CDS of all identified *Petunia LSH* genes shows that 9 of 11 *PhLSH* genes, including *PhLSH1, PhLSH2, PhLSH3b, PhLSH4, PhLSH7a, PhLSH7b, PhLSH10b, PhLSH10b,* and *PhLSH10c,* are grouped with orthologous genes from *P. axillaris* (Fig. 3), suggesting that *PhLSH* genes might mainly originate from *P. axillaris*. Further alignment and comparison of the coding sequences of these *LSHs* genes indicates that 5 of 11 *PhLSHs* (*PhLSH4, PhLSH7a, PhLSH7b, PhLSH10a, PhLSH10c*) are identical to their orthologous *PaLSHs* (Additional file 6); moreover, *PhLSH4, PhLSH7a, PhLSH7b* and *PhLSH10c* are different at least for one nucleotide from *PiLSHs*, *PintLSHs* and *PeLSHs*, confirming they come from *P. axillaris.* The CDS of *PhLSH10a* is the same as those of *PiLSH10a*, *PintLSH10a,* and *PeLSH10a,* but its 3’UTR sequence is identical to that of *PaLSH10a* and different from those of *PeLSH10a* and *PintLSH10a* (data not shown), indicating that *PhLSH10a* also come from *P. axillaris*. The coding sequences of *PhLSH1* and *PhLSH10b* contain only one-nucleotide differences compared to those of *PaLSH1* and *PaLSH10b* but at least four-nucleotide differences in comparison to the orthologs from other three species, suggesting that *PhLSH1* and *PhLSH10b* may also originate from *P. axillaris*. The sequences of *PhLSH3a* and *PhLSH5* show substantial differences in all four species but have more similarity to the orthologs of *P. exserta*, indicating that they may have originated from *P. exserta.*

### ALOG domains and motifs of PhLSHs

**Fig. 4:**
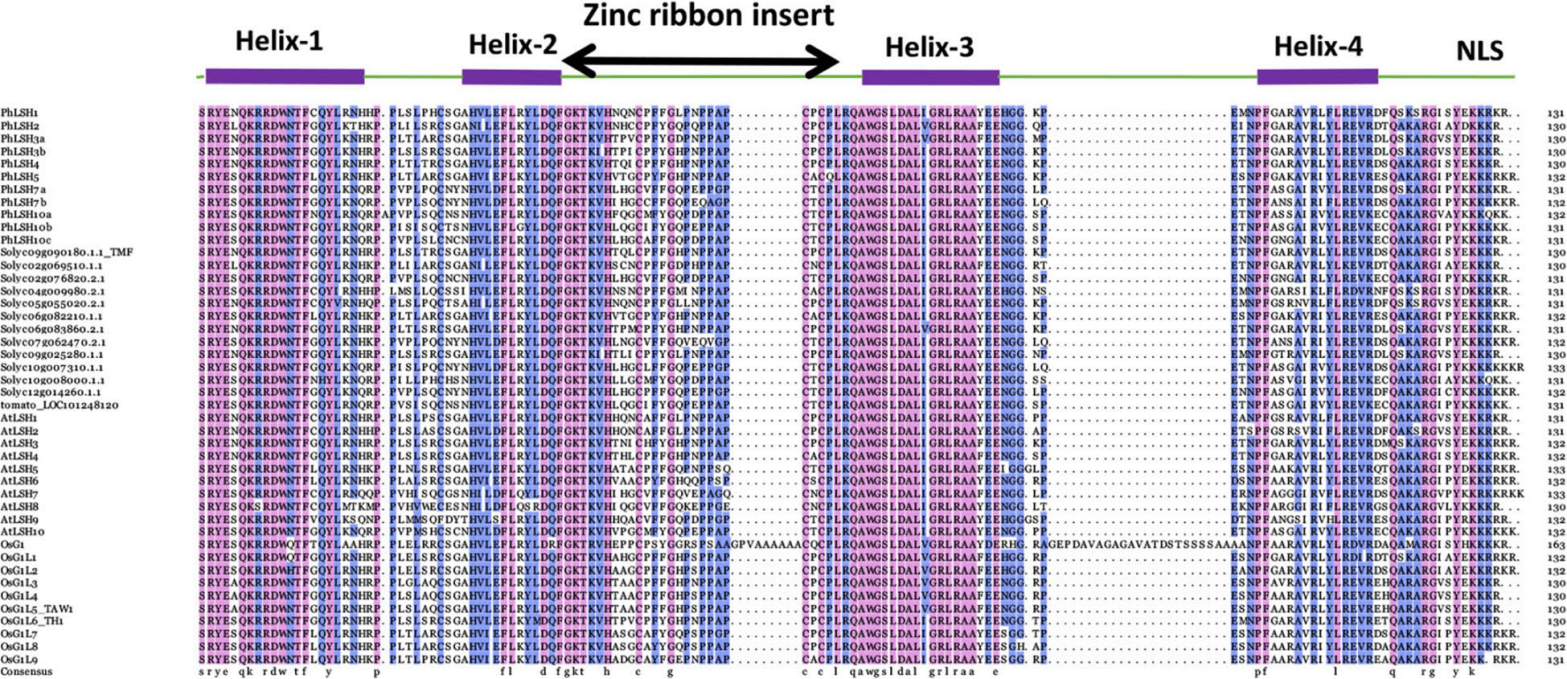
Characteristics of the ALOG domains. Multiple alignments of the highly conserved ALOG domains (130 aa) of 11 PhLSH proteins, 13 SlyLSH proteins, 10 AtLSH proteins, and 10 OsG1-like proteins. The conserved ALOG domain included a zinc ribbon insert structure and NLS. Sequences were colored based on 75% consistency. The absolutely conserved residues are shown in light purple

**Fig. 5:**
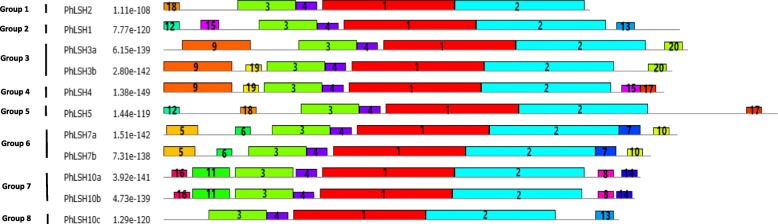
Motifs in the PhLSH proteins predicted by MEME

To investigate the sequence conservation and divergence of petunia ALOG members, multiple sequence alignment was carried out and conserved motifs were identified in the 11 PhLSH proteins. The results demonstrate that all PhLSH proteins contain a conserved ALOG domain in the middle region, which consists of four helixes with a zinc ribbon insert and a nuclear location signal (NLS) sequence (Fig. 4), however, the similarity of the N- and C-terminal sequences is very low (Additional file 7), which is consistent with previous reports in other species [﻿29﻿, 31, 32]. Motif analysis shows that a total of 20 motifs are present in PhLSH proteins and the number of motifs in each protein varies from 5 to 8 (Fig. 5, Additional file 8). Motifs 1–4 constitute the ALOG domain and exist in all PhLSHs, while other motifs show group- or member-specific distribution. For instance, motif 9 appears only in the Group 3 and Group 4 proteins PhLSH3a/3b/4; motifs 5–7 and 10 only exist in the Group 6 proteins PhLSH7a/7b; motifs 8, 11, 14, and 16 are specific to the Group 7 proteins PhLSH10a and PhLSH10b.

### Spatial expression of *PhLSHs*

**Fig. 6:**
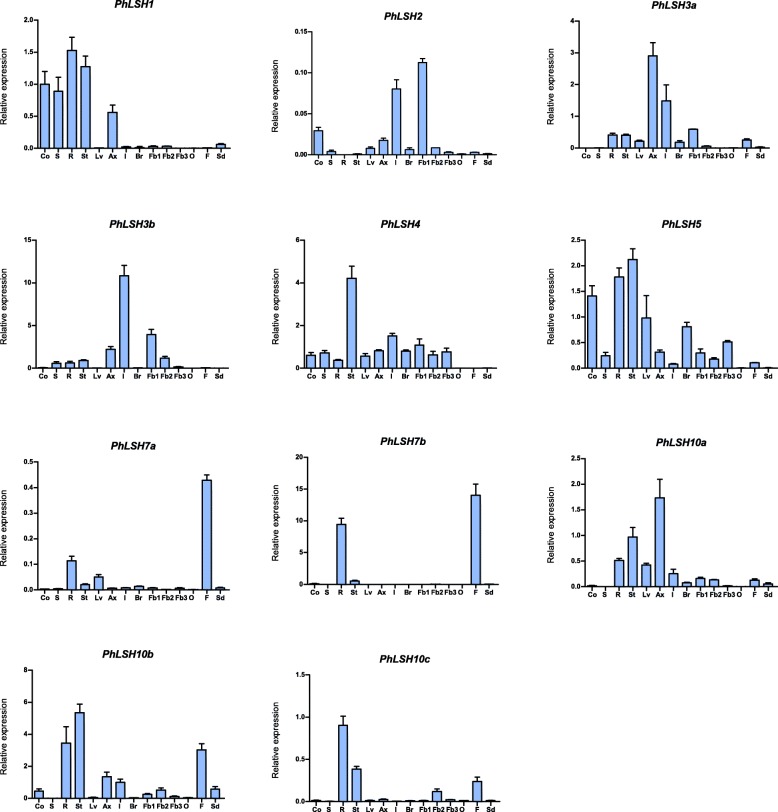
Expression analysis of *PhLSHs* by qPCR. Co, cotyledons; S, seedlings; R, roots; St, stems; Lv, leaves; Br, bracts; Ax, axillary buds; I, inflorescences; Fb1–3, flower buds (0.1, 0.5, and 6 cm, respectively); O, ovaries; F, fruits, and Sd, seeds. *PhEF1α* was used as reference gene for the transcript level. For each tissue, three biological replicates were used to calculated the mean values ± SD (standard deviation) with the 2^−ΔΔCT^ method. The expression of *PhLSH1* in Co was used as calibrator

Expression analysis of *PhLSHs* in different organs by qPCR shows that *PhLSH* genes were expressed in different patterns among various tissues (Fig. 6, Additional file 9). Among them, the most specific expression is the members belonging to Group 6, *PhLSH7a* and *PhLSH7b*, both of which are only significantly expressed in roots and fruits, whereas the expression level of *PhLSH7b* is much higher (about 100 folds) than that of *PhLSH7a*. In contrast, *PhLSH4* and *PhLSH5* show more ubiquitous expression in most tissues with the highest expression in stems. Different from *PhLSH4*, however, which is not expressed in fruits and lower expressed in cotyledons and roots, *PhLSH5* has low expression in fruits and relatively high expression in cotyledons and roots. *PhLSH3a* and *PhLSH3b* belong to the same group but have differential expression profile. *PhLSH3a* shows the highest expression level in axillary buds followed by inflorescences, while *PhLSH3b* has the highest expression in inflorescences followed by young flower buds, and that the expression level of *PhLSH3b* was much higher than that of *PhLSH3a*. Similarly, the expression patterns of the Group 7 members (*PhLSH10a* and *PhLSH10b*) are different. *PhLSH10a* is mainly expressed in axillary buds followed by stems, while *PhLSH10b* is mainly expressed in roots, stems, seedling, and fruits. *PhLSH10c*, a close paralogs of *PhLSH10a/10b*, is only significantly expressed in roots. *PhLSH1* has very low expression levels in all reproductive organs, but with high expression levels in the vegetative organs (except for leaves). *PhLSH2* is the member with the lowest expression levels compared to other genes, mainly expressed in inflorescences and young flower buds.

### Function of PhLSH7a and PhLSH7b in Arabidopsis

**Fig. 7:**
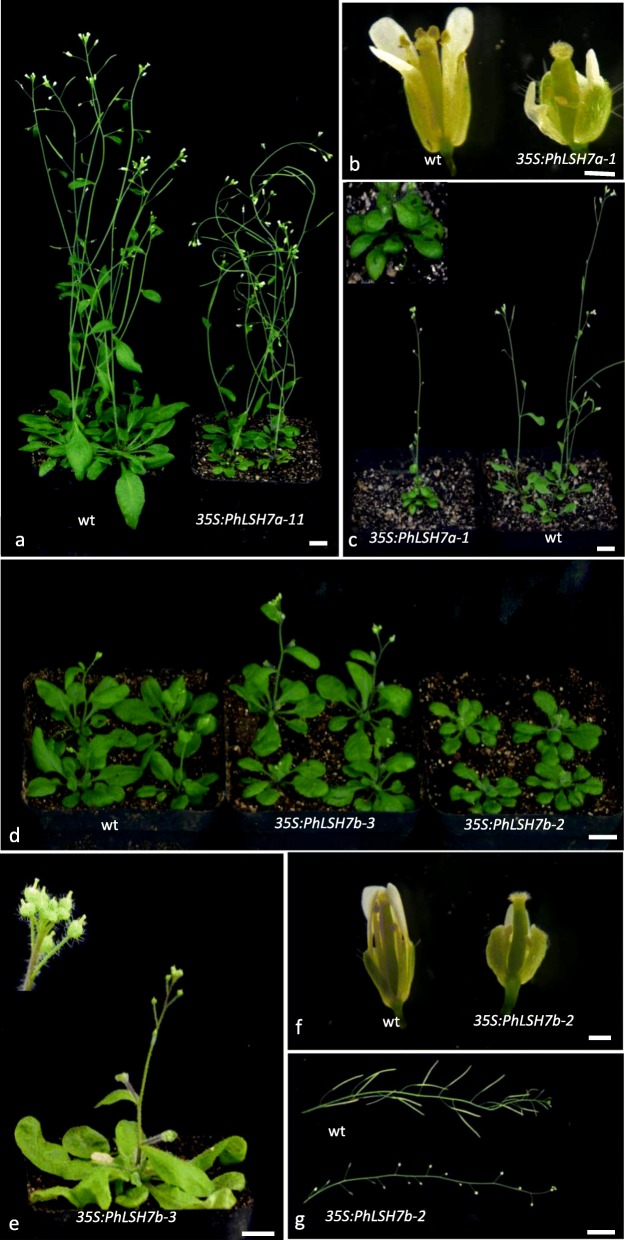
Phenotypes of the *35S:PhLSH7a* and *35S:PhLSH7b* transgenic *Arabidopsis* plants. **a** The *35S:PhLSH7a-11* transgenic plants (right) show small and round leaves compared to the wild-type (left). **b** A flower of wild type (wt, left) and *35S:PhLSH7a-1* (right) show deformed flowers of *35S:PhLSH7a* transgenic plants in which one petal and one sepal were removed. **c** The *35S:PhLSH7a-1* transgenic plants (left) show small and round leaves compared to wt (right). The picture in the upper left is a close-up of small and round leaves. **d** The *35S:PhLSH7b* transgenic plants (*35S:PhLSH7b-2* and *35S:PhLSH7b-3*) show small round leaves compared to wt (left), and *35S:PhLSH7b-2* (right) is late flowering compared to wt (left) and *35S:PhLSH7b-3* (middle). **e** The flowers of *35S:PhLSH7b-3* show the deformed flowers with abnormal petals and exposed pistil out of an unopened flower. The picture in the upper left is a close-up of deformed flowers. **f** A flower of wt (left) and *35S:PhLSH7b-2* (right) show deformed flowers of *35S:PhLSH7b* transgenic plants in which one petal and one sepal were removed. **g** Fruits of *35S:PhLSH7b-2* (right) show a sterile phenotype compared to wt (left). Bars: 10 mm (a, c, d, e, and g), 0.5 mm (b and f)

**Fig. 8:**
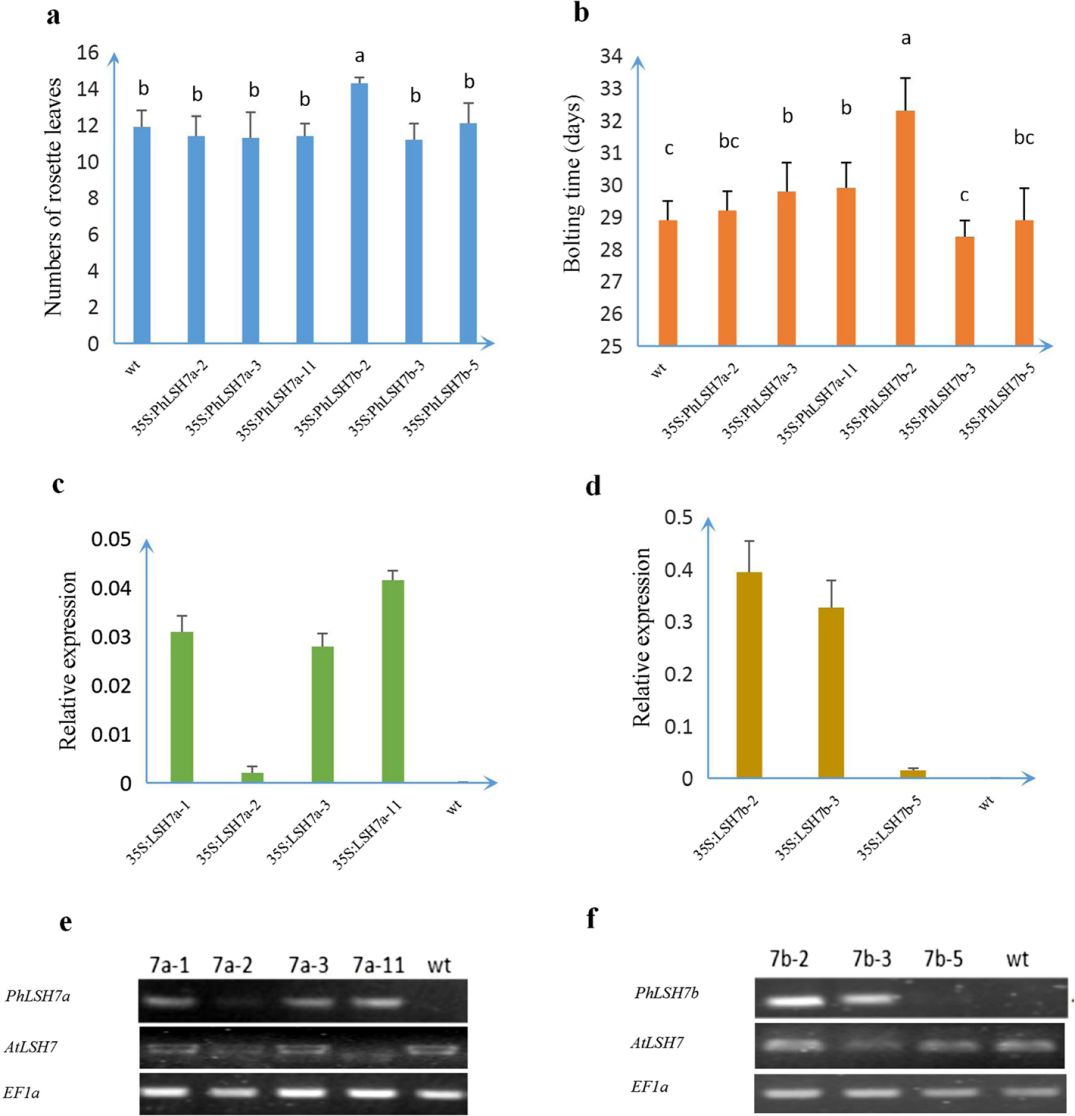
Phenotype and RT-PCR analysis of transgenic *Arabidopsis* plants. **a** Comparison of rosette leaves (RL) in wt and transgenic lines shows that *35S:PhLSH7b-2* contains more rosette leaves than others. The letters indicate the result of statistical differences. **b** Comparison of flowering time (FT) in wt and transgenic lines shows that *35S:PhLSH7b-2* is later flowering than others. The letters indicate the result of statistical differences. **c, d** qPCR analysis shows the expression level of transgenes in wt and transgenic *Arabidopsis* lines. *35S:PhLSH7a-11* has higher expression level than other *35S:PhLSH7a* lines (c); *35S:PhLSH7b-2* has higher expression level than other *35S:PhLSH7b* lines (d). The phenotype changes were largely related to the expression level of transgenes (Fig. 7)**. e, f** Semi quantitative RT-PCR analysis shows the expression level of transgenes in wt and transgenic *Arabidopsis* lines consistent with the results of qPCR. There is no any interference with the two endogenous genes, *AtLSH7/8* (*AtLSH8* cannot be detected, so its semi-quantitative results are not displayed)

Over-expression of *PhLSH7a* and *PhLSH7b* in *Arabidopsis* shows that both genes have remarkable effects on the vegetative and reproductive growth of transgenic plants. There were significant phenotypic changes in *PhLSH7a/7b*-overexpressing lines, including small round leaves (Fig. 7a, c, d), late flowering (Fig. 7d), and deformed flowers such as abnormal petals and exposed pistil out of an unopened flower (Fig. 7b, e, f). In particular, there is no progeny can be obtained from the lines with strong phenotype (*35S:PhLSH7a-1* and *35S:PhLSH7b-2*) by self-pollinating (Fig. 7c, g). However, when the wild-type plants were used as pollen donors to cross with *35S:PhLSH7b-2*, some small siliques and few seeds can be harvested, which suggests that the male and female fertility of transgenic plants were both significantly decreased, and infertility of the *35S:PhLSH7b-2* line may mainly due to the sterile pollen. Unfortunately, we failed to obtain seeds from *35S:PhLSH7a-1* even after pollinated with wild-type pollen. Other *35S:PhLSH7a* transgenic lines with weaker phenotype still show small round leaves (Fig. 7a). In addition, the progenies of *35S:PhLSH7b-2* show late flowering compared to wild type and other transgenic lines. RT-PCR and qPCR showed that phenotype changes were largely related to the expression level of exogenous transgene instead of endogenous gene (Fig. 8; Additional file 10).

## Discussion

### Evolution of *ALOG* gene family

ALOG family plays important roles in plant morphogenesis and organ development. The ALOG domain has been proposed to be derived from the XerC/D-like recombinases of a new category of DIRS-1-like retroposons and was probably acquired during the evolution of streptophyte-clade plants [10]. According to recent studies, the evolution of the ALOG domain is dominated by lineage-specific duplication and these expansions seem to have occurred after the separation of monocot and dicot lineages [10, 31]. The evolutionary history of the ALOG family over plant diversification, however, still remains largely unclear. Recently, Xiao et al. [31] carried out a comprehensive phylogenetic analysis of the ALOG family with genes from basal land plants, monocots, and eudicots, which showed that the genes from different plant lineages clustered independently. Among the grass lineage, three clades were present: *GrassALOG1* including rice *G1* (*OsG1*) and *OsG1L1/2*, *GrassALOG2* including *OsG1L3/4/5*, and *GrassALOG3* including *OsG1L6/7/8/9*; while the *ALOG* genes of eudicot lineage were further divided into four clades: *euALOG1* (*AtLSH1/2/3/4*), *euALOG2* (without *Arabidopsis* gene), *euALOG3* (*AtLSH7/8/9/10*), and *euALOG4* (*AtLSH5/6*). In our phylogenetic analysis, an unrooted tree was constructed with the ALOG domains of 73 proteins from 9 representative species belonging to different evolutionary clades. The results indicated these proteins could be divided into five clades (Fig. 2), which is similar to the results reported by Iyer and Aravind [10] but different from the results of Xiao et al. [31]. Phylogenetic analysis with seven species including a basal land plant species (*Physcomitrella patens*), two eudicots (*Arabidopsis* and *Populus trichocarpa*) and four grasses divided the ALOG proteins into six distinct clusters [﻿29﻿]: cluster A, B, and C are *Physcomitrella* and monocot-specific, including OsG1L1/2, OsG1 and OsG1L3/4/5/6, respectively; cluster D and F are eudicot-specific, including AtLSH7/8/9/10 and AtLSH1/2/3/4, respectively; cluster E contains proteins of both grasses (OsG1L7/8/9/10) and eudicots (AtLSH5/6). These results indicate that the evolutionary history of the ALOG proteins appears to be complicated, and we need more sequences and studies to clarify it.

According to our phylogenetic tree, petunia *LSH* genes can be divided into four clades consisting of 8 groups (Fig. 2): Group 1 contains *PhLSH2* and orthologs from basal angiosperm, monocots, and core eudicots, but not *Arabidopsis* and basal eudicots; Group 2 contains *PhLSH1* and orthologs from *P. aphrodite* and core eudicots; Group 3 contains *PhLSH3a/3b* and orthologs of *P. aphrodite* and core eudicots; Group 4 contains *PhLSH4* and orthologs of eudicots; Group 5 contains *PhLSH5* and orthologs of all other species except for *A. coerulea*; Group 6 contains *PhLSH7a/7b* and orthologs of other core eudicots; Group 7 contains *PhLSH10a/10b* and orthologs from all other eudicots except for *N. nucifera*; Group 8 contains *PhLSH10c* and orthologs from all other eudicots except for *Arabidopsis*. This result is consistent with the classification of *LSH* genes in crucifer [﻿30﻿] and indicates that the ALOG family has undergone multiple times of independent duplication and/or loss events during the evolution of angiosperm. Furthermore, species-specific late expansions of *ALOG* genes in Solanaceae can be found from the phylogenetic tree, such as *PhLSH7a/7b* and *PhLSH10a/10b* gene pairs. In *P. inflata*, we found three *PhLSH5* orthologs that have complete identical coding sequences (Additional file 2), which may also come from very recent duplication in this species.

### Expression and functions of ALOG proteins in petunia

Currently, the functions of *ALOG* genes are only available for a few members in *Arabidopsis*, rice, and tomato. It is generally believed that gene function is closely related to their expression characteristics. So, we investigated the spatial expression profile of petunia *LSHs*. Consequently, *PhLSH* genes showed divergent expression patterns with different tissue specificity (Fig. 6), suggesting that they may have diverse functions in regulating the development of these organs.

*PhLSH1* is proved to be the orthologs of *Arabidopsis AtLSH1*/*2* genes (Fig. 2). In *Arabidopsis*, *AtLSH1* is expressed in cotyledons, hypocotyls, shoot apices, and roots (especially in lateral root primordium), and it is functionally dependent on phytochrome to mediate light regulation of seedling development [4]. Similar to *AtLSH1*, *PhLSH1* is expressed mainly in the vegetative organs including seedlings, cotyledons, roots, stems, and axillary buds (Fig. 6), implying it may have comparable functions to *AtLSH1*. Different from the situation in *Arabidopsis*, which contains two very close paralogs *AtLSH1* and *AtLSH2* that may result in functional redundancy, petunia contains only one gene in this group, so functional characterization of *PhLSH1* will be easier. *PhLSH2* has no orthologs in *Arabidopsis* or functionally known group members, and that it has very low expression levels in all tissues compared to other *PhLSH* genes, so its function maintains elusive. It may attribute to the low expression for the fact that no *PhLSH2* orthologs was identified in *P. integrifolia* and *P. exserta*.

*PhLSH3a/3b* and *PhLSH4* are the orthologs of *AtLSH3* and *AtLSH4*, respectively. In the same clade, it also includes rice *TAW1* and tomato *TMF*, two functional characterized genes. *AtLSH3* and *AtLSH4* are both expressed in boundary cells of various lateral organs including cotyledons, leaves, and floral organs, which may inhibit organ differentiation in the boundary regions [7, 15]. In contrast, petunia *PhLSH3a/3b* and *PhLSH4* show different expression patterns, in which *PhLSH3a* mainly expressed in axillary buds followed by inflorescences, *PhLSH3a* mainly in inflorescences, and *PhLSH4* mainly in stems (Fig. 6), suggesting that petunia *PhLSH3a/3b/4* genes may have different functions from their orthologs in *Arabidopsis*. *TMF*, an orthologous gene of *PhLSH4* in tomato, is expressed predominantly in the shoot apex, with high expression in vegetative stages of the primary shoot meristems (PSM) and the sympodial vegetative meristems (SYM), slightly decreased expression in the reproductive transition meristems, and weak expression in the floral meristems (FM), sympodial inflorescence meristems (SIM), and young leaves [8]. *TMF* synchronizes the flowering transition and flower formation by timing *AN* activation, which has a key role in determining simple versus complex inflorescences. Similar to *TMF*, rice *TAW1* gene were also expressed predominantly in meristems, including the shoot apical meristems (SAM), axillary meristems, primary inflorescence meristems (IM), and branch meristems (BM), to regulate inflorescence architecture through the suppression of meristems phase transition [6]. In petunia, single flower other than multi-flowered inflorescence is produced, so it is very curious to know the functions of *PhLSH3a/3b* and *PhLSH4*.

*PhLSH5* is a member of the clade IV that contains genes from most species including basal angiosperm, monocots, basal eudicots, and core eudicots. No member has been functionally characterized in this group, however, almost ubiquitous expression pattern of *PhLSH5* and wide distribution of its orthologs in plants imply that it might play important and multiple roles in plant vegetative and reproductive development. *PhLSH7a* and *PhLSH7b*, two co-orthologs of *Arabidopsis AtLSH7/8* genes, show similar tissue-specific expression patterns, predominantly expressed in young fruits, suggesting they may play roles in fruit development. In accordance with this, over-expression of *PhLSH7a* or *PhLSH7b* in *Arabidopsis* has a strong impact on the fertility and fruit development of the transgenic plants. The *35S:PhLSH7a* and *35S:PhLSH7b* transgenic *Arabidopsis* plants also show comparable phenotypic changes during vegetative growth such as small round leaves [Fig. 7]. The highly similar expression patterns and protein function of the *PhLSH7a*/*7b* gene pair indicate they may have redundant functions. *PhLSH10a* and *PhLSH10b* belong to the same group with high homology of protein sequences that contains identical motifs (Fig. 5), but have different expression patterns (Fig. 6), suggesting they may be functionally divergent genes. *PhLSH10c* gene probably is involved in regulating root development based on its predominant expression in roots [Fig. 6].

## Conclusions

In this study, the *ALOG* family genes (*LSHs*) were identified from four wild *Petunia* species and the hybrid petunia line W115 at genome or transcriptome scales. Based on the analysis of gene structure, phylogenetic relationship, expression pattern and function, it is concluded that the *ALOG* family has complicated evolution history and diverse expression patterns and functions, and petunia *PhLSH7a/7b* genes have significant functions in plant development, especially in fruit development. This work provides an important foundation for further elucidating the biological function of *LSH* genes in petunia.

## Methods

### Plant materials and grow conditions

*Petunia hybrida* line W115 (MD) and *Arabidopsis thaliana* (Col-0) were used as the plant materials in this study and the seeds was originally obtained from Department of Molecular Cell Biology, VU-University, The Netherlands (gifts by prof. Ronald Koes). Plant materials were grown in greenhouse under a light condition of 16 h light/8 h dark and a luminous intensity of 170 μmol·m^− 2^·s^− 1^, with a humidity of 75% and a temperature of 22–25 °C.

### Identification of *ALOG* genes from petunia species

The protein sequences of *Arabidopsis* and rice *ALOG* genes were downloaded from NCBI. The sequence data of *P. inflata* and *P. axillaris* were retrieved from Sol Genomics Network (https://solgenomics.net/) [26]. Two approaches were used to identify ALOG family genes in petunia. First, the ALOG protein sequences identified previously in *Arabidopsis* and rice were used as queries to search the genome and transcriptome datasets of *P. inflata* and *P. axillaris* [26, 27, ﻿33﻿] using the tBLASTx (translated nucleotide to translated nucleotide) program with default parameters. Second, the HMM profile for the DUF640 domain (PF04852) obtained from the Pfam database was used to search the annotated petunia proteomes. The obtained sequences were merged to remove redundancy and examined for the presence of the ALOG domain at the NCBI CD search (https://www.ncbi.nlm.nih.gov/Structure/cdd/wrpsb.cgi).

The *ALOG* genes from *P. integrifolia*, *P. exserta* and *P. hybrida* were recognized by BLAST searching the transcriptome databases at NCBI [27, ﻿34﻿] using the nucleotide sequences of the obtained *P. inflata* and *P. axillaris ALOG* genes as queries.

### Cloning of *ALOG* genes from petunia W115

To further confirm the identified genes, full-length genomic DNA sequence and complete CDS of *P. hybrida* line W115 *ALOG* genes were amplified using gene-specific primers. Mixture of different tissues was used to extract total RNA with RNA pure Total RNA Kit (Aidlab, China). 2 μg of RNA was used to synthesize the cDNA using cDNA synthesis Kit (Takara, Japan) with Oligo dT Primer and Random 6 mers. Then 2 μl of cDNA dilution (1:20) was added in PCR system with the 2 × High-Fidelity Master Mix DNA Polymerase (Tsingke, China). The products of PCR were purified with Axyprep DNA Gel Extraction Kit (Axygen, USA), and then cloned into pMD18-T (Takara, Japan) followed by heat shock into *E. coli* DH5α. For each gene, three positive clones were picked for sequencing (Augct, China).

### Multiple sequence alignments and phylogenetic analysis

MEGA 6.0 [35] was used to align the CDS and protein sequences of petunia *ALOG* genes with the ClustalW program. To figure out the evolutionary scenario of the *ALOG* gene family in angiosperms, ALOG protein sequences from 9 representative species belonging to different angiosperm clades, including *Amborella trichopoda* as a basal angiosperm, two monocots (*Phalaenopsis aphrodite* and *Oryza sativa*), two basal eudicots (*Nelumbo nucifera* and *Aquilegia coerulea*), and four core eudicots (*P. hybrida* and *Solanum lycopersicum* from the asterids; *Vitis vinifera* and *Arabidopsis thaliana* from the rosids), were used for phylogenetic analysis. Besides the 11 petunia genes cloned in this study (Table 1) and 20 previously identified *ALOG* genes in *Arabidopsis* [4] and rice [5], we identified 4 ALOG proteins from *A. trichopoda* and *A. coerulea*, respectively, 6 from *N. nucifera*, 7 from *P. aphrodite*, 8 from *V. vinifera*, and 13 from *S. lycopersicum* based on their genome databases (Additional file 4). Multiple alignments of these protein sequences were performed by the MUSCLE program of MEGA 6.0 [35], and the conserved ALOG domains (about 130 aa) were applied to construct phylogenetic tree using the Maximum Likelihood (ML) method with JTT + G model and 1000 replicates of bootstrap. The JTT + G model with the lowest BIC scores is considered the best model in MEGA6 model selection. Phylogenetic tree of the *LSH* genes from five *Petunia* species was constructed with the full-length coding sequences using ML method with the GTR + G + I model (the best model) and 1000 bootstrap replicates.

### Gene structure and motif analysis

The exon-intron distribution of the *PaLSHs, PiLSHs* and *PhLSHs* genes were analyzed using the Gene Structure Display Server 2.0 (http://gsds.cbi.pku.edu.cn/) [36]. The CDD in NCBI was used to identify conserved domains of the *PhLSH* proteins with default parameters [37]. Motifs of PhLSH proteins were predicted by the MEME 4.12.0 (http://meme-suite.org/tools/meme) with parameter settings as follows: the minimum motif width = 6, the maximum motif width = 50, and the maximum number of motifs = 20 [38].

### Quantitative RT-PCR

Various samples were collected from W115 petunia, including germinating seeds (sow in petri dish for 3 d), cotyledons (sow in petri dish for 7 d), 5-euphylla seedlings; roots, stems, leaves, and axillary buds (collected from flowering plants); bracts, inflorescences, flower buds (0.1 cm, 0.5 cm, and 6 cm), ovaries (without pollination), and young fruits (5 d after pollination). All samples were kept in liquid nitrogen immediately and hold in − 80°C freezer until they were used.

RNA extraction and quantitative real-time PCR (qPCR) was performed with the reported method [39]. *PhEF1α* was used as a reference gene [40]. Three biological replicates were applied to calculate the mean values ± SD (standard deviation) with the 2^−ΔΔCT^ method [41]. The expression of *PhLSH1* in Co was used as calibrator. Primer5.0 was used to design the primers with a product size of 150–250 bp. The specificity of all primers was tested by RT-PCR.

### Plasmid construction and *Arabidopsis* transformation

The pMD-18 T vectors containing *PhLSH7a* or *PhLSH7b* genes was double digested by *BamHI* and *SalI* (Takara, Japan), then the products were ligated into the pCAMBIA2300 vector to obtain *35S:PhLSH7a* and 35S*:PhLSH7b* construction. Both PCR and double enzyme digestion were used to confirm the constructed plasmids that were electrically transferred into *Agrobacterium tumefaciens* GV3101. Floral dip method was applied for *Arabidopsis* (Col-0) transformation as previously reported [42].

### Phenotype and transgene expression analysis

Both T1 and T_2_ generation lines were chosen to record morphological characteristics such as the number of rosette leaves and flowering time. Severely sterile lines were artificially pollinated with wild-type pollen to collect seeds. RT-PCR was applied to analyze the expression level of transgenes and endogenous genes in *Arabidopsis* as previously reported [﻿42﻿]. ANOVA (analysis of variance) was used to analyze the significant differences with Tukey’s post-test (*P* < 0.05).

## Supplementary information

**Additional file 1. ***PaLSH* genes in the *P. axillaris* genome. ^a^ Sequence ID related to the database from https://solgenomics.net/organism/Petunia_axillaris/genome (v1.6.2) [26]. ^b^ The transcripts correspond to the TSA (Transcriptome Shotgun Assembly) database of *P. axillaris* in NCBI [27]. ‘/’ indicates no transcript was identified.

**Additional file 2. ***PiLSH* genes in the *P. inflata* genome. ^a^ Sequence ID related to the database from https://solgenomics.net/organism/Petunia_axillaris/genome (v1.0.1) [26]. ^b^ The transcripts were identified by nucleotide BLAST search of the TSA (Transcriptome Shotgun Assembly) database of *P. integrifolia* (GBRV) and *P. integrifolia Subsp. inflata* (GBDS) in the NCBI [﻿33﻿]. ‘/’ indicates no orthologous transcript was identified.

**Additional file 3. **Primers used in this study for cloning *PhLSH* CDS, qRT-PCR and transgene line RT-PCR.

**Additional file 4. **Sequence details in the phylogenetic analysis. A total of 73 protein sequences were included in the phylogenetic analysis: 11 sequences from *Petunia hybrida*, 10 sequences from *Arabidopsis*, 10 from rice, 8 from *Vitis vinifera*, 4 from *Amborella trichopoda*, 7 from *Phalaenopsis aphrodite*, 6 from *Nelumbo nucifera*, 4 from *Aquilegia coerulea* and 13 from *Solanum lycopersicum*.

**Additional file 5. ***PeLSH* and *PintLSH* genes in the transcriptome database. These genes were isolated by nucleotide BLAST search of the TSA (Transcriptome Shotgun Assembly) database of *P. integrifolia* and *P. exserta* [﻿29﻿]. ‘–’ means no transcripts was found. Partial indicated that the ORF sequences were incomplete.

**Additional file 6. **Variations in the CDS and amino acids between *PhLSH*s and their orthologs in *P. axillaris*, *P. inflata*, *P. integrifolia* and *P. exserta*. ‘/’ means no gene was isolated; ‘-’ means no protein was translated.

**Additional file 7.** Multiple alignments of 11 PhLSH proteins. The similarity of the N- and C-terminal sequences is very low. Sequences were colored based on 75% consistency. The absolutely conserved residues are shown in light purple.

**Additional file 8.** Detailed characteristics of the motifs in the PhLSH proteins.

**Additional file 9. **Heat map demonstrating the expression of *PhLSH*s in different tissues using averaged log2 relative expression value. The expression range is shown in color based on a scale. Sd, seeds; Co, cotyledons; S, seedlings; R, roots; St, stems; Lv, leaves; Ax, axillary buds; Br, bracts; I, inflorescences; Fb1–3, flower buds (0.1 cm, 0.5 cm, and 6 cm); O, ovaries and F, fruits. *PhEF1α* was the reference gene for the transcript level.

**Additional file 10. **Phenotype analysis of the *35S:PhLSH7a* and *35S:PhLSH7b* transgenic *Arabidopsis* plants. Values are the mean ± SD (*n* = 32). The letters indicate the result of statistical differences (*P* < 0.05).

## Data Availability

The genome sequences of *P. axillaris* and *P. inflata* used for identifying the *LSH* genes in current study were located in Sol Genomics Network (https://solgenomics.net/organism/Petunia_axillaris/genome and https://solgenomics.net/organism/Petunia_inflata/genome, respectively) [26]. The transcriptome databases used to identify *LSH* genes of *P. integrifolia*, *P. exserta* and *P. axillaris* were available in NCBI under accessions GBRU01000000, GBRT01000000, and GBRV01000000, respectively [27]. The transcriptome databases of *P. inflata* were available in NCBI under accessions GBDS00000000, GBDR00000000, and GBDQ00000000 [﻿33﻿]. The transcriptome sequences of *P. hybrida* ‘Mitchell’ was located in Sol Genomics Network (https://solgenomics.net/) [﻿34﻿]. The sequences information of *PhLSHs* was uploaded to the NCBI (accession number MK179267- MK179276).

## Abbreviations

ALOG: *Arabidopsis* LSH1 and *Oryza* G1
CDS: Coding sequence
*LSH*: *Light-dependent short hypocotyls*
ML: Maximum likelihood
NLS: Nuclear localization signal
ORF: Open reading frame
qPCR: Quantitative real-time PCR
RT-PCR: Reverse transcription PCR
SAM: Shoot apical meristems
TFs: Transcription factors
UTR: Untranslated region
