# Supplementary material for: Genome-wide identification and characterization of the ALOG gene family in Petunia
Source: BMC Plant Biol. 2019 Dec 30;19:600. doi: 10.1186/s12870-019-2127-x (PMC6937813; doi:10.1186/s12870-019-2127-x)
Supplement: Supplementary file 1 — Additional file 1. PaLSH genes in the P. axillaris genome. a Sequence ID related to the database from https://solgenomics.net/organism/Petunia_axillaris/genome (v1.6.2) [26]. b The transcripts correspond to the TSA (Transcriptome Shotgun Assembly) database of P. axillaris in NCBI [27]. ‘/’ indicates no transcript was identified. [file 12870_2019_2127_MOESM1_ESM.doc]

| **Gene name** | **Sequence ID of genomic DNA^a^** | **Regions of exons (bp)** | **Gene length (bp)** | **ORF length (bp)** | **Protein length (aa)** | **Transcripts in TSA database^b^** |
| --- | --- | --- | --- | --- | --- | --- |
| *PaLSH1* | Peaxi162Scf00015 | 3120282-3120059 (224), 3119517-3119157 (361) | 1126 | 585 | 194 | GBRU01025616.1 |
| *PaLSH2* | Peaxi162Scf00195 | 317299-316817 (483) | 483 | 483 | 160 | / |
| *PaLSH3a* | Peaxi162Scf00013 | 1151092-1151391 (300), 1151756-1152052 (297) | 961 | 597 | 198 | GBRU01067667.1 |
| *PaLSH3b* | Peaxi162Scf00134 | 1569764-1570339 (576) | 576 | 576 | 191 | GBRU01048844.1 |
| *PaLSH4* | Peaxi162Scf01265 | 217243-217809 (567) | 567 | 567 | 188 | GBRU01043717.1 |
| *PaLSH5* | Peaxi162Scf00390 | 894500-894386(115), 894288-893717 (572) | 784 | 687 | 228 | GBRU01034412.1 |
| *PaLSH7a* | Peaxi162Scf00692 | 632515-631934 (582) | 582 | 582 | 193 | GBRU01037400.1 |
| *PaLSH7b* | Peaxi162Scf00111 | 1565467-1564913 (555) | 555 | 555 | 184 | GBRU01008455.1 |
| *PaLSH10a* | Peaxi162Scf00666 | 440809-440270 (540) | 540 | 540 | 179 | GBRU01069500.1 |
| *PaLSH10b* | Peaxi162Scf00469 | 285814-286347 (534) | 534 | 534 | 177 | GBRU01065759.1 |
| *PaLSH10c* | Peaxi162Scf01106 | 187267-187782 (516) | 516 | 516 | 171 | GBRU01027341.1 |
