# Supplementary material for: Genome-wide identification and characterization of the ALOG gene family in Petunia
Source: BMC Plant Biol. 2019 Dec 30;19:600. doi: 10.1186/s12870-019-2127-x (PMC6937813; doi:10.1186/s12870-019-2127-x)
Supplement: Supplementary file 2 — Additional file 2. PiLSH genes in the P. inflata genome. a Sequence ID related to the database from https://solgenomics.net/organism/Petunia_axillaris/genome (v1.0.1) [26]. b The transcripts were identified by nucleotide BLAST search of the TSA (Transcriptome Shotgun Assembly) database of P. integrifolia (GBRV) and P. integrifolia Subsp. inflata (GBDS) in the NCBI [﻿33﻿]. ‘/’ indicates no orthologous transcript was identified. [file 12870_2019_2127_MOESM2_ESM.doc]

| **Gene name** | **Sequence ID of genomic DNA^a^** | **Regions of exons (bp)** | **Gene length (bp)** | **ORF length (bp)** | **Protein length (aa)** | **Transcripts in**  **TSA database^b^** |
| --- | --- | --- | --- | --- | --- | --- |
| *PiLSH1* | Peinf101Scf00056 | 928583-928806 (224); 929397-929763 (367) | 1181 | 591 | 196 | GBRV01064068.1 |
| *PiLSH2* | Peinf101Scf00793 | 708562-709044 (483) | 483 | 483 | 160 | / |
| *PiLSH3a* | Peinf101Scf18795 | 1558-1244 (315); 796-524 (273) | 1035 | 588 | 195 | GBRV01060955.1 |
| *PiLSH3b* | Peinf101Scf00879 | 2291157-2290582 (576) | 576 | 576 | 191 | GBRV01101478.1 |
| *PiLSH4* | Peinf101Scf00672 | 882260-881688 (573) | 573 | 573 | 190 | GBRV01086025.1 |
| *PiLSH5* | Peinf101Scf08825 | 233-347 (115); 446-1026 (581)  8155-8269 (115); 8368-8958 (581) | 794  794 | 696  696 | 231  231 | GBDS01003230.1  GBRV01050246.1 |
|  | Peinf101Scf02200 | 109217-109331 (115); 109430-110010 (581) | 794 | 696 | 231 |  |
| *PiLSH7a* | Peinf101Scf00132 | 1297982-1298566 (585) | 585 | 585 | 194 | GBRV01082383.1 |
| *PiLSH7b* | Peinf101Scf00665 | 327790-328344 (555) | 555 | 555 | 184 | GBRV01034733.1 |
| *PiLSH0a* | Peinf101Scf00305 | 152813-152274 (540) | 540 | 540 | 179 | GBRV01101542.1 |
| *PiLSH10b* | Peinf101Scf01267 | 17001-16468 (534) | 534 | 534 | 177 | GBRV01088501.1 |
| *PiLSH10c* | Peinf101Scf00543 | 1793182-1793697 (516) | 516 | 516 | 171 | GBRV01055637.1 |
