# Supplementary material for: Genome-wide identification and characterization of the ALOG gene family in Petunia
Source: BMC Plant Biol. 2019 Dec 30;19:600. doi: 10.1186/s12870-019-2127-x (PMC6937813; doi:10.1186/s12870-019-2127-x)
Supplement: Supplementary file 3 — Additional file 3. Primers used in this study for cloning PhLSH CDS, qRT-PCR and transgene line RT-PCR. [file 12870_2019_2127_MOESM3_ESM.doc]

| **a** |  |
| --- | --- |
| **Primers** | **Sequence (5’ to 3’)** |
| *PhLSH1(fw)*  *PhLSH1(rev)* | TTACTTCTAGCACAATAAGGTCTA  TCACATTCTCCTAAATTACTTACTAC |
| *PhLSH2(fw)*  *PhLSH2(rev)* | TACAAACACATCAACACACAGC  CCTAGACGGCTGGCTATCAT |
| *PhLSH3a(fw)*  *PhLSH3a(rev)* | ATGAAATCCTAGAAAGATTTG  ACGTAACTAATATGATAAGAGATGATGAAG |
| *PhLSH3b(fw)*  *PhLSH3b(rev)* | AAGTGATTGATCCAAAGAATTAAAGC  CCGCAAAGTGCTAGGAAATATG |
| *PhLSH4(fw)*  *PhLSH4(rev)* | TTCTTGTACTTTCTTTCTCTTCCA  GAACATACCATAATACGACATTGAT |
| *PhLSH5(fw)*  *PhLSH5(rev)* | GATGACATTCAATTCATTCCTGAC  CTGAACTAGAAGAGAGATGAAACAA |
| *PhLSH7a(fw)*  *PhLSH7a(rev)* | CCATTCCATGTCTCCTCAGT  GAAATTCAAAGCAATCAAGAAGA |
| *PhLSH7b(fw)*  *PhLSH7b(rev)* | GTATGATGTCAAGTGAGCAAAG  GCTCTTGAGCAAAGCCAAA |
| *PhLSH10a(fw)*  *PhLSH10a(rev)* | TATGTCAAGTGATAGAGGCAAAG  ACAAGCACGAGGTTCTTCA |
| *PhLSH10b(fw)*  *PhLSH10b(rev)* | CACCATCCACTTCAATTCTACTAA  AGCAGGCACTAGGCATCA |
| *PhLSH10c(fw)*  *PhLSH10c(rev)* | CCTTTATTCATCCTCCTTTCTCTT  GCAGCAGCATCTTCTTATCTAA |

**b**

| **Primers** | **Sequence (5’ to 3’)** | **Description** |
| --- | --- | --- |
| *PhLSH1(fw)*  *PhLSH1(rev)* | TTTTACTTCTAGCACAATAAGGTCT  AGAAGATGGTATGATTGCACTGTTG | qRT-PCR |
| *PhLSH2(fw)*  *PhLSH2(rev)* | GTGCAAGAGCTGTGAGGTTGTATTT  TAGACGGCTGGCTATCATGGTAA | qRT-PCR |
| *PhLSH3a(fw)*  *PhLSH3a(rev)* | TAGTTACGATAAGAAAAAACGGAAA  ACGTAACTAATATGATAAGAGATGATGA | qRT-PCR |
| *PhLSH3b(fw)*  *PhLSH3b(rev)* | ATCAAGAAGTGATTGATCCAAAGAA  AGATGATGATGCTATGTTACTGTTG | qRT-PCR |
| *PhLSH4(fw)*  *PhLSH4(rev)* | GATTCTCCAAACCTTTCCATCCACA  TTAGGGTCGTAGTTGGAGAAGAAGA | qRT-PCR |
| *PhLSH5(fw)*  *PhLSH5(rev)* | GTCAGAGTATCCGACCCTAACAGTA  CACTGACCGTTATTGTGGATGGACC | qRT-PCR |
| *PhLSH7a(fw)*  *PhLSH7a(rev)* | CCATTCCATGTCTCCTCAGT  GCTAATACAATAGCAGCTGATGATT | qRT-PCR and transgene RT-PCR |
| *PhLSH7b(fw)*  *PhLSH7b(rev)* | TCCATTTGCAAATAGTGCTATAAGA  GCTCTTGAGCAAAGCCAAA | qRT-PCR and transgene RT-PCR |
| *PhLSH10a(fw)*  *PhLSH10a(rev)* | TTCCAGTTCTGCAGGGTTCTTC  ACAAGCACGAGGTTCTTCA | qRT-PCR |
| *PhLSH10b(fw)*  *PhLSH10b(rev)* | TCTTAGGGAAGTGAAAGAGTGTCAA  AGCAGGCACTAGGCATCA | qRT-PCR |
| *PhLSH10c(fw)*  *PhLSH10c(rev)* | ATTCATCCTCCTTTCTCTTTTTGTA  AAAGTGATAATGGAACTGGTGGTCT | qRT-PCR |
| *PhEF1a(fw)*  *PhEF1a(rev)* | CCTGGTCAAATTGGAAACGG  CAGATCGCCTGTCAATCTTGG | qRT-PCR and RT-PCR control |
| *AtEF1α(fw)*  *AtEF1α(rev)* | GCAAGATGGATGCCACTACCC  AGTGGGAGACGAAGGGGCT | transgene RT-PCR control |
