# Supplementary material for: Genome-wide identification and characterization of the ALOG gene family in Petunia
Source: BMC Plant Biol. 2019 Dec 30;19:600. doi: 10.1186/s12870-019-2127-x (PMC6937813; doi:10.1186/s12870-019-2127-x)
Supplement: Supplementary file 4 — Additional file 4. Sequence details in the phylogenetic analysis. A total of 73 protein sequences were included in the phylogenetic analysis: 11 sequences from Petunia hybrida, 10 sequences from Arabidopsis, 10 from rice, 8 from Vitis vinifera, 4 from Amborella trichopoda, 7 from Phalaenopsis aphrodite, 6 from Nelumbo nucifera, 4 from Aquilegia coerulea and 13 from Solanum lycopersicum. [file 12870_2019_2127_MOESM4_ESM.doc]

| [Abbreviation](../../../../C:/Program%20Files%20(x86)/Youdao/Dict/8.4.0.0/resultui/html/index.html#/javascript:;) | Species | Sequence ID |
| --- | --- | --- |
| *AqcoLSH4a* | *Aquilegia coerulea* | PIA36400.1 |
| *AqcoLSH4b* | *Aquilegia coerulea* | PIA45395.1 |
| *AqcoLSH10a* | *Aquilegia coerulea* | PIA42195.1 |
| *AqcoLSH10b* | *Aquilegia coerulea* | PIA34055.1 |
| *ViviLSH1* | *Vitis vinifera* | XP_002267312.1 |
| *ViviLSH2* | *Vitis vinifera* | XP_002271024.1 |
| *ViviLSH3* | *Vitis vinifera* | XP_010652376.1 |
| *ViviLSH4* | *Vitis vinifera* | XP_010650097.1 |
| *ViviLSH6* | *Vitis vinifera* | XP_002278283.1 |
| *ViviLSH7* | *Vitis vinifera* | XP_002285447.1 |
| *ViviLSH10a* | *Vitis vinifera* | XP_002280666.1 |
| *ViviLSH10b* | *Vitis vinifera* | XP_010655519.1 |
| *NenuLSH4a* | *Nelumbo nucifera* | XP_010246797.1 |
| *NenuLSH4b* | *Nelumbo nucifera* | XP_010262340.1 |
| *NenuLSH6a* | *Nelumbo nucifera* | XP_010257059.1 |
| *NenuLSH6b* | *Nelumbo nucifera* | XP_010264562.1 |
| *NenLSH10a* | *Nelumbo nucifera* | XP_010277024.1 |
| *NenuLSH10b* | *Nelumbo nucifera* | XP_010279129.1 |
| *AmtrG1* | *Amborella trichopoda* | XP_006847409.1 |
| *AmtrG1L1a* | *Amborella trichopoda* | XP_006857299.1 |
| *AmtrG1L1b* | *Amborella trichopoda* | XP_011624010.1 |
| *AmtrG1L9* | *Amborella trichopoda* | XP_020524645.1 |
| *PheqG1L1* | *Phalaenopsis equestris* | XP_020586337.1 |
| *PheqG1L3* | *Phalaenopsis equestris* | XP_020575793.1 |
| *PheqG1L6a* | *Phalaenopsis equestris* | XP_020594691.1 |
| *PheqG1L6b* | *Phalaenopsis equestris* | XP_020584905.1 |
| *PheqG1L7* | *Phalaenopsis equestris* | XP_020596878.1 |
| *PheqG1L9a* | *Phalaenopsis equestris* | XP_020585912.1 |
| *PheqG1L9b* | *Phalaenopsis equestris* | XP_020582092.1 |
| *SolyLSH1a* | *Solanum lycopersicum* | Solyc05g055020.3 |
| *SolyLSH1b* | *Solanum lycopersicum* | Solyc04g009980.3 |
| *SolyLSH2* | *Solanum lycopersicum* | Solyc02g069510.1 |
| *SolyLSH3a* | *Solanum lycopersicum* | Solyc06g083860.2 |
| *SolyLSH3b* | *Solanum lycopersicum* | Solyc09g025280.1 |
| *TMF* | *Solanum lycopersicum* | Solyc09g090180.1 |
| *SolyLSH5* | *Solanum lycopersicum* | Solyc06g082210.1 |
| *SolyLSH7a* | *Solanum lycopersicum* | Solyc10g007310.1 |
| *SolyLSH7b* | *Solanum lycopersicum* | Solyc07g062470.3 |
| *SolyLSH7c* | *Solanum lycopersicum* | Solyc12g014260.1 |
| *SolyLSH10a* | *Solanum lycopersicum* | Solyc10g008000.1 |
| *SolyLSH10b* | *Solanum lycopersicum* | Solyc07g055065.1 |
| *SolyLSH10c* | *Solanum lycopersicum* | Solyc02g076820.3 |
