# Supplementary material for: Genome-wide identification and characterization of the ALOG gene family in Petunia
Source: BMC Plant Biol. 2019 Dec 30;19:600. doi: 10.1186/s12870-019-2127-x (PMC6937813; doi:10.1186/s12870-019-2127-x)
Supplement: Supplementary file 5 — Additional file 5. PeLSH and PintLSH genes in the transcriptome database. These genes were isolated by nucleotide BLAST search of the TSA (Transcriptome Shotgun Assembly) database of P. integrifolia and P. exserta [﻿29﻿]. ‘–’ means no transcripts was found. Partial indicated that the ORF sequences were incomplete. [file 12870_2019_2127_MOESM5_ESM.docx]

| **Gene name** | **Sequence ID** | **The length of ORFs (bp)** | **The length of putative proteins (amino acid)** |
| --- | --- | --- | --- |
| *PeLSH1* | [GBRT01043791.1](https://www.ncbi.nlm.nih.gov/nuccore/GBRT01043791) | 591 | 196 |
| *PeLSH2* | - | - | - |
| *PeLSH3a* | [GBRT01043764.1](https://www.ncbi.nlm.nih.gov/nuccore/GBRT01043764) | 585 | 194 |
| *PeLSH3b* | [GBRT01048614.1](https://www.ncbi.nlm.nih.gov/nuccore/GBRT01048614) | 576 | 191 |
| *PeLSH4* | [GBRT01032520.1](https://www.ncbi.nlm.nih.gov/nuccore/GBRT01032520) | 570 | 189 |
| *PeLSH5* | [GBRT01021378.1](https://www.ncbi.nlm.nih.gov/nuccore/GBRT01021378) | 702 | 233 |
| *PeLSH7a* | [GBRT01023975.1](https://www.ncbi.nlm.nih.gov/nuccore/GBRT01023975) | 582 | 193 |
| *PeLSH7b* | [GBRT01023398.1](https://www.ncbi.nlm.nih.gov/nuccore/GBRT01023398) | 552 | 183 |
| *PeLSH10a* | [GBRT01049512.1](https://www.ncbi.nlm.nih.gov/nuccore/GBRT01049512) | 540 | 179 |
| *PeLSH10b* | [GBRT01045359.1](https://www.ncbi.nlm.nih.gov/nuccore/GBRT01045359) | 534 | 177 |
| *PeLSH10c* | [GBRT01020198.1](https://www.ncbi.nlm.nih.gov/nuccore/GBRT01020198) | 516 | 171 |
| *PintLSH1* | [GBRV01064068.1](https://www.ncbi.nlm.nih.gov/nuccore/GBRV01064068) | 591 | 196 |
| *PintLSH2* | - | - | - |
| *PintLSH3a* | [GBRV01060955.1](https://www.ncbi.nlm.nih.gov/nuccore/GBRV01060950) | 597 | 198 |
| *PintLSH3b* | [GBRV01101478.1](https://www.ncbi.nlm.nih.gov/nuccore/GBRV01101478) | 576 | 191 |
| *PintLSH4* | [GBRV01086025.1](https://www.ncbi.nlm.nih.gov/nuccore/GBRV01086025) | >485 | >160 |
| *PintLSH5* | [GBRV01050246.1](https://www.ncbi.nlm.nih.gov/nuccore/GBRV01050246) | 696 | 231 |
| *PintLSH7a* | [GBRV01082383.1](https://www.ncbi.nlm.nih.gov/nuccore/GBRV01082383) | >537 | >179 |
| *PintLSH7b* | [GBRV01034733.1](https://www.ncbi.nlm.nih.gov/nuccore/GBRV01034733) | 555 | 184 |
| *PintLSH10a* | [GBRV01101542.1](https://www.ncbi.nlm.nih.gov/nuccore/GBRV01101542) | 540 | 179 |
| *PintLSH10b* | [GBRV01088501.1](https://www.ncbi.nlm.nih.gov/nuccore/GBRV01088501) | 534 | 177 |
| *PintLSH10c* | [GBRV01055637.1](https://www.ncbi.nlm.nih.gov/nuccore/GBRV01055637) | 516 | 171 |
