# Supplementary material for: Genome-wide identification and characterization of the ALOG gene family in Petunia
Source: BMC Plant Biol. 2019 Dec 30;19:600. doi: 10.1186/s12870-019-2127-x (PMC6937813; doi:10.1186/s12870-019-2127-x)
Supplement: Supplementary file 6 — Additional file 6. Variations in the CDS and amino acids between PhLSHs and their orthologs in P. axillaris, P. inflata, P. integrifolia and P. exserta. ‘/’ means no gene was isolated; ‘-’ means no protein was translated. [file 12870_2019_2127_MOESM6_ESM.docx]

| **Genes of *P. hybrida* line W115** | **Orthologs in *P. axillaris*** | **Nucleotide differences** | **Amino acid variations** | **Orthologs in *P. inflata*** | **Nucleotide differences** | **Amino acid variations** | **Orthologs in *P. axillaris*** | **Nucleotide differences** | **Amino acid variations** | **Orthologs in *P. inflata*** | **Nucleotide differences** | **Amino acid variations** |
| --- | --- | --- | --- | --- | --- | --- | --- | --- | --- | --- | --- | --- |
| *PhLSH1* | *PaLSH1* | 1 | 0 | *PiLSH1* | 18 | 14 | *PintLSH1* | 14 | 14 | *PeLSH1* | 8 | 14 |
| *PhLSH2* | *PaLSH2* | 0 | 0 | *PiLSH2* | 4 | 1 | *PintLSH2* | / | / | *PeLSH2* | / | / |
| *PhLSH3a* | *PaLSH3a* | 28 | 9 | *PiLSH3a* | 64 | 21 | *PintLSH3a* | 31 | 10 | *PeLSH3a* | 16 | 5 |
| *PhLSH3b* | *PaLSH3b* | 1 | 0 | *PiLSH3b* | 3 | 1 | *PintLSH3b* | 2 | 1 | *PeLSH3b* | 1 | 1 |
| *PhLSH4* | *PaLSH4* | 0 | 0 | *PiLSH4* | 9 | 3 | *PintLSH4* | >7 | >4 | *PeLSH4* | 3 | 0 |
| *PhLSH5* | *PaLSH5* | 21 | 7 | *PiLSH5* | 33 | 13 | *PintLSH5* | 39 | 12 | *PeLSH5* | 17 | 10 |
| *PhLSH7a* | *PaLSH7a* | 0 | 0 | *PiLSH7a* | 9 | 2 | *PintLSH7a* | >9 | - | *PeLSH7a* | 1 | 0 |
| *PhLSH7b* | *PaLSH7b* | 0 | 0 | *PiLSH7b* | 4 | 2 | *PintLSH7b* | 4 | 2 | *PeLSH7b* | 2 | 0 |
| *PhLSH10a* | *PaLSH10a* | 0 | 0 | *PiLSH10a* | 2 | 1 | *PintLSH10a* | 0 | 0 | *PeLSH10a* | 0 | 0 |
| *PhLSH10b* | *PaLSH10b* | 1 | 0 | *PiLSH10b* | 9 | 1 | *PintLSH10b* | 11 | 1 | *PeLSH10b* | 4 | 0 |
| *PhLSH10c* | *PaLSH10c* | 0 | 0 | *PiLSH10c* | 9 | 2 | *PintLSH10c* | 8 | 2 | *PeLSH10c* | 2 | 0 |
