# Supplementary material for: Genome-wide identification and characterization of the ALOG gene family in Petunia
Source: BMC Plant Biol. 2019 Dec 30;19:600. doi: 10.1186/s12870-019-2127-x (PMC6937813; doi:10.1186/s12870-019-2127-x)

motif1


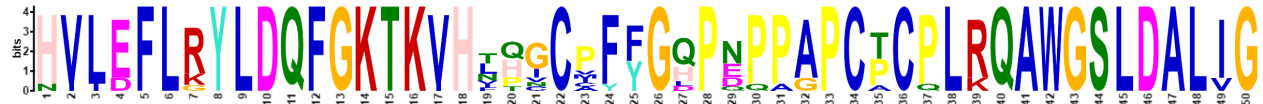


motif2


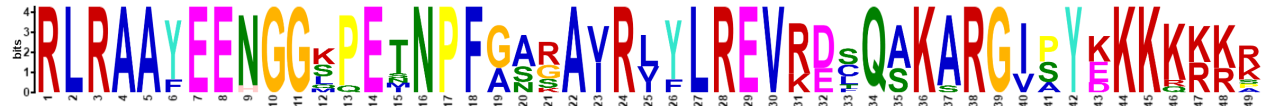


motif3


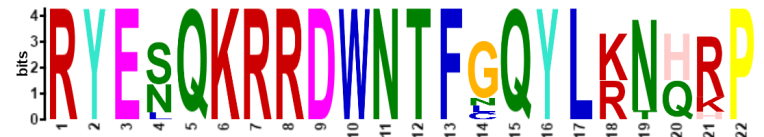


motif4


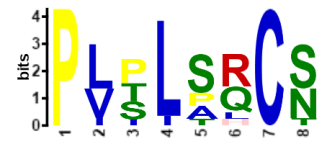


motif5


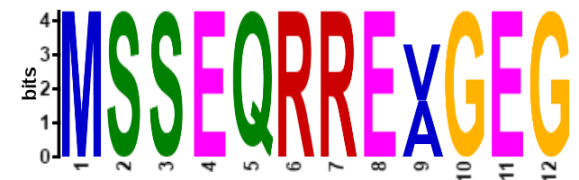


motif6


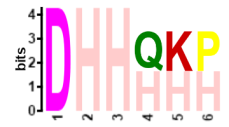


motif7


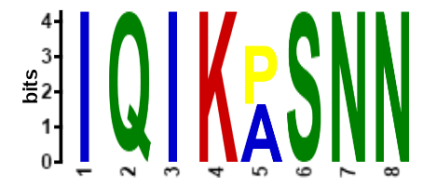


motif8


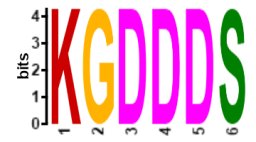


motif9


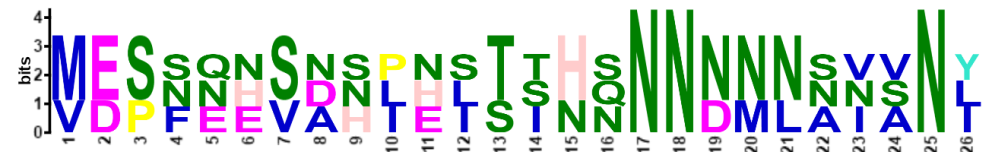


motif10


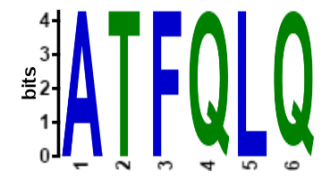


motif11


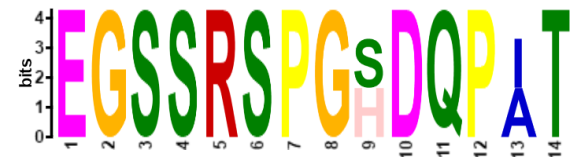


motif12


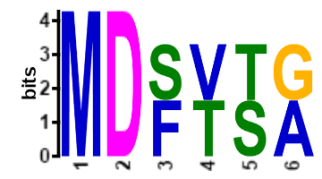


motif13


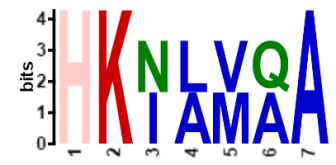


motif14


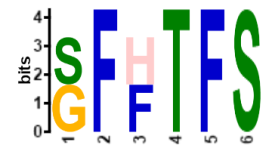


motif15


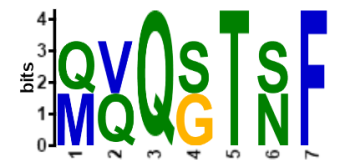


motif16


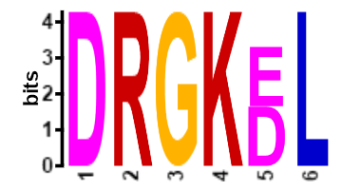


motif17


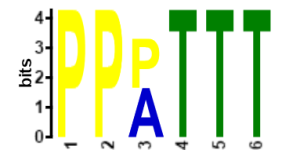


motif18


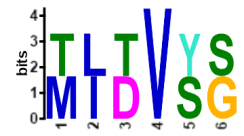


motif19

**
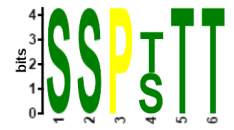
**

motif20


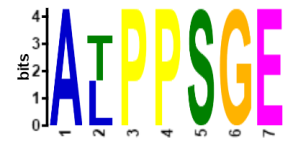

Supplement: Supplementary file 8 — Additional file 8. Detailed characteristics of the motifs in the PhLSH proteins. [file 12870_2019_2127_MOESM8_ESM.docx]
