# Supplementary figures and images for: Genome-wide identification and characterization of the ALOG gene family in Petunia
Source: BMC Plant Biol. 2019 Dec 30;19:600. doi: 10.1186/s12870-019-2127-x (PMC6937813; doi:10.1186/s12870-019-2127-x)

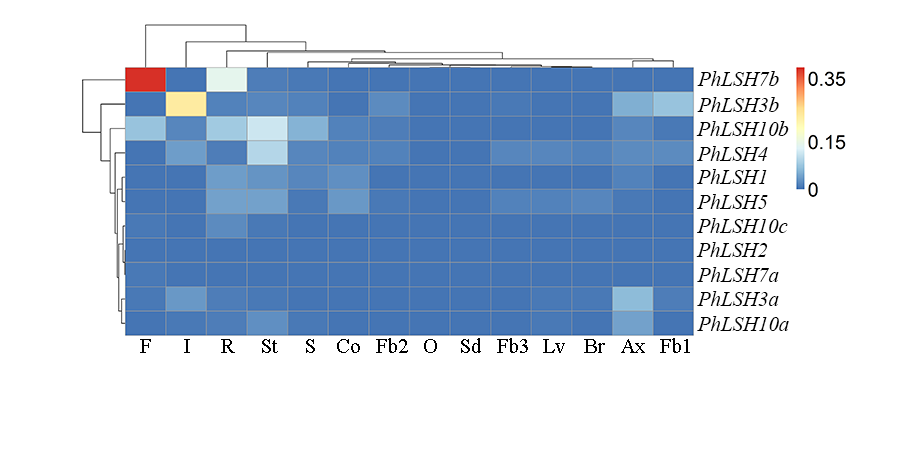

Supplement: Supplementary file 9 — Additional file 9. Heat map demonstrating the expression of PhLSHs in different tissues using averaged log2 relative expression value. The expression range is shown in color based on a scale. Sd, seeds; Co, cotyledons; S, seedlings; R, roots; St, stems; Lv, leaves; Ax, axillary buds; Br, bracts; I, inflorescences; Fb1–3, flower buds (0.1 cm, 0.5 cm, and 6 cm); O, ovaries and F, fruits. PhEF1α was the reference gene for the transcript level. [file 12870_2019_2127_MOESM9_ESM.doc]
