# Supplementary material for: Genome-wide identification and characterization of the ALOG gene family in Petunia
Source: BMC Plant Biol. 2019 Dec 30;19:600. doi: 10.1186/s12870-019-2127-x (PMC6937813; doi:10.1186/s12870-019-2127-x)
Supplement: Supplementary file 10 — Additional file 10. Phenotype analysis of the 35S:PhLSH7a and 35S:PhLSH7b transgenic Arabidopsis plants. Values are the mean ± SD (n = 32). The letters indicate the result of statistical differences (P < 0.05). [file 12870_2019_2127_MOESM10_ESM.doc]

| **Lines** | **Rosette leaves (numbers)** | **Cauline leaves (numbers)** | **Bolting time (days)** |
| --- | --- | --- | --- |
| wt | 11.9±0.9 b | 2.1±0.5 | 28.9±0.6 c |
| *35S:PhLSH7a-2* | 11.7±1.1 b | 2.1±0.6 | 29.2±0.6 bc |
| *35S:PhLSH7a-3* | 11.3±1.4 b | 2.5±0.9 | 29.8±0.9 b |
| *35S:PhLSH7a-11* | 11.4±0.7 b | 2.5±0.5 | 29.9±0.8 b |
| *35S:PhLSH7b-2* | 14.3±0.3 a | 2.4±0.5 | 32.3±1.0 a |
| *35S:PhLSH7b-3* | 11.2±0.9 b | 2.5±0.6 | 28.4±0.5 c |
| *35S:PhLSH7b-5* | 12.1±1.1 b | 2.4±0.9 | 28.9±1.0 bc |
