# Supplementary material for: Genome-wide identification and characterization of the ALOG gene family in Petunia
Source: BMC Plant Biol. 2019 Dec 30;19:600. doi: 10.1186/s12870-019-2127-x (PMC6937813; doi:10.1186/s12870-019-2127-x)
Supplement: Supplementary file 10 [file 12870_2019_2127_MOESM7_ESM.doc]

```
PhLSH1      ..........MDFVTAQGNFSGNN.....MVQGTN.......FLPNSAIIPSSAPPLSRYENQKRRDWNTFCQYLRNHHP.PLSLPHCSGAHVLEFLRYLDQFGKTKVHNQNCPFFGLPNPPAPC  102
PhLSH2      ...............MLDVYSTSS.....ISENFS..........SPTAQPPSLPTASRYELQKRRDWNTFGQYLKTHKP.PLILSRCSGANILEFLKYLDQFGKTKVHNHCCPFYGQPQPPAPC   94
PhLSH3a     .MATFT.SEVESSNHSANIHSTTH.....QNNDNNSIVNLGSSTTLSSSSSSTVSTSSRYENQKRRDWNTFGQYLKNHRP.PLTLARCSGAHVLEFLRYLDQFGKTKVHTPVCPFYGDPNPPAPC  117
PhLSH3b     .MDSF.....ENSNH.LEITSNSN.....NNNNNN.......SNIASSSSSSPSTTSSRYENQKRRDWNTFGQYLKNHRP.PLSLSRCSGAHVLEFLRYLDQFGKTKIHTPICPFYGHPNPPAPC  105
PhLSH4      .MEPNQ....EVDSPNLSIHNNNN.....MLAVAN..........YSASSSSPTTTLSRYENQKRRDWNTFGQYLRNHRP.PLTLTRCSGAHVLEFLRYLDQFGKTKVHTQICPFFGHPNPPAPC  104
PhLSH5      .MDSTSGVRVSDPNSSGGGGGGAG.....EGEGPSTITVSGQAIDTRGGSTTSAAPPSRYESQKRRDWNTFLQYLRNHKP.PLTLARCSGAHVIEFLKYLDQFGKTKVHVTGCPYFGHPNPPAPC  118
PhLSH7a     MMS.......SEQRREVGE.GSSSKSSAAIVLAPS.........DHHHHHQPAPPQLSRYESQKRRDWNTFGQYLKNQRP.PVPLPQCNYNHVLDFLRYLDQFGKTKVHLHGCVFFGQPEPPGPC  107
PhLSH7b     .MS.......SEQRREAGE.GSSS.....LVVPP............DHHQKPPSPQLSRYESQKRRDWNTFGQYLKNQRP.PVPLSQCNYNHVLDFLRYLDQFGKTKVHIHGCCFFGQPEQAGPC   98
PhLSH10a    .MS.......SDRGKDLAAEGSSR........SP.............GHDQP..ITPSRYESQKRRDWNTFNQYLRNQRPAPVPLSQCNSNHVLEFLRYLDQFGKTKVHFQGCMFYGQPDPPAPC   94
PhLSH10b    .MSN......IDRGKELVE.GSSR........SP.............GSDQP..ATLSRYESQKRRDWNTFGQYLRNQRP.PISISQCTSNHVLEFLGYLDQFGKTKVHLQGCIFYGQPEPPAPC   93
PhLSH10c    ............MAKELSE.GSSK......NIAP......................LSRYESQKRRDWNTFGQYLKNQRP.PVPLSLCNCNHVLEFLRYLDQFGKTKVHLHGCAFFGQPDPPAPC   83
Consensus                                                            srye qkrrdwntf qyl    p p     c       fl yldqfgktk h   c   g p    pc

PhLSH1      PCPLRQAWGSLDALIGRLRAAYEEHGGKPEMNPFGARAVRLFLREVRDFQSKSRGISYEKKRKRS...STNKHKIAVAAAAA.TLCAGQTNTSNVV...................  194
PhLSH2      PCPLKQAWGSLDALVGRLRAAFEENGGQPEINPFGARAVRLYLREVRDTQAKARGIAYDKKKRRK...S...........................................  160
PhLSH3a     PCPLRQAWGSLDALVGRLRAAFEENGGMPETNPFGARAVRLYLREVRDLQSKARGVSYDKKKRKR...PPPSQPLATPP.......SGEA........................  197
PhLSH3b     PCPLRQAWGSLDALIGRLRAAYEENGGKPETNPFGARAVRLYLREVRDLQSKARGVSYEKKKRKR...PPPSHQLPSPPPPA.LPPSGET........................  191
PhLSH4      PCPLRQAWGSLDALIGRLRAAYEENGGKPETNPFGARAVRLYLREVRDSQAKARGISYEKKKRKK...PSSQQQQSTSFPP...PTTTSS........................  188
PhLSH5      ACQLKQAWGSLDALIGRLRAAYEENGGKPESNPFGAKAVRIYLREVRESQAKARGIPYEKKKRKR...STSNLATSGSGTAVGDGDSGGGDGGGNVSGGGGAVVSPPATTTTTTTT  230
PhLSH7a     TCPLRQAWGSLDALIGRLRAAYEENGGLPETNPFASGAIRVYLREVRDSQSKARGIPYKKKKKRRIQIKASNNDNPAAATFQ..LQSS..........................  193
PhLSH7b     TCPLRQAWGSLDALIGRLRAAYEENGGLQETNPFANSAIRIFLREVRDSQAKARGIPYKKKKKKRIQIKPSNN.NEVPATFQ..LQSS..........................  183
PhLSH10a    TCPLRQAWGSLDALIGRLRAAYEENGGSPETNPFASSAIRVYLREVKECQAKARGVAYKKKQKKP..SPSNHKGDDDSSSAG.FFTFS..........................  179
PhLSH10b    TCPLRQAWGSLDALIGRLRAAYEENGGSPETNPFASGAIRVYLREVKECQAKARGIPYKKKKKKAG.TSSESKGDDDSTS...FHTFS..........................  177
PhLSH10c    TCPLRQAWGSLDALIGRLRAAYEENGGSPENNPFGNGAIRLYLREVKECQAKARGIPYKKKKKRKLNNPSKAIGAAASDQHKNLMQAS..........................  171
Consensus    c l qawgsldal grlraa ee gg  e npf   a r  lrev   q k rg  y kk
```
